# Pan-Cancer Analysis and Experimental Validation of SOX4 as a Potential Diagnosis, Prognosis, and Immunotherapy Biomarker

**DOI:** 10.3390/cancers15215235

**Published:** 2023-10-31

**Authors:** Xinna Deng, Yashu Wang, Hao Guo, Qian Wang, Shuting Rao, Haijiang Wu

**Affiliations:** 1 Departments of Oncology, Hebei General Hospital, Shijiazhuang 050057, China; dengxinna001@163.com (X.D.); 13739741321@163.com (H.G.); wanghairui12345@163.com (Q.W.); 15909416438@163.com (S.R.); 2 Department of Pathology, Hebei Medical University, Shijiazhuang 050011, China; medicine147@sina.com; 3 Department of Internal Medicine, Hebei Medical University, Shijiazhuang 050011, China

**Keywords:** SOX4, pan-cancer, hepatocellular carcinoma, lenvatinib, drug resistance

## Abstract

**Simple Summary:**

Data show that the global cancer burden continues to grow. It is estimated that there were 19.3 million new cancer cases and nearly 10 million cancer deaths worldwide in 2020. Therefore, a comprehensive analysis is of great significance for the accurate diagnosis and effective treatment of tumors. This study aims to visualize the expression, survival, mutation, methylation, ceRNA network, and immune and prognostic models, as well as to explore the potential role of SOX4 in different tumor types and specific LIHCs using different online tools. In addition, we also confirm that SOX4 may be related to lenvatinib resistance in HCC patients through in vitro experiments. Knockdown of SOX4 can significantly inhibit the proliferation of HCC cells. Finally, this study highlights the key roles of SOX4 in the diagnosis and prognosis of tumors, especially in LIHC, and its potential as a promising therapeutic target for tumors.

**Abstract:**

Introduction: SOX4 plays an important role in tumorigenesis and cancer progression. The role of SOX4 in pan-cancer and its underlying molecular mechanism in liver hepatocellular carcinoma (LIHC) are not fully understood. In this study, a comprehensive analysis and experimental validation were performed to explore the function of SOX4 across tumor types. Methods: Raw data in regard to SOX4 expression in malignant tumors were downloaded from the TCGA and GTEx databases. The expression levels, prognostic values, genetic mutation, and DNA promoter methylation of SOX4 across tumor types were explored via systematic bioinformatics analysis. The ceRNA regulatory network, immune characteristics, and prognostic models were analyzed in LIHC. Finally, we conducted in vitro experiments including Western blotting, cell proliferative assay, trypan blue staining, and fluorescence microscopy to further explore the function of SOX4 in LIHC. Results: SOX4 expression was significantly upregulated in 24 tumor types. SOX4 expression level was strongly associated with unfavorable prognoses, genetic mutations, and DNA methylation levels across different tumor types. Especially in LIHC, LINC00152/hsa-miR-139-3p/SOX4 was identified as a crucial ceRNA network. Moreover, this study also provides insight into the roles of SOX4 expression in immune cell infiltration, macrophage polarization, immune subtype, molecular subtype, and immunomodulators, as well as the tumor immune microenvironment (TIME)-related prognosis, in LIHC. The study established six favorable prognostic models to predict LIHC prognosis based on the SOX4-associated genes. Finally, lenvatinib treatment can increase the expression of SOX4 in hepatocellular carcinoma cells and lead to drug resistance. Silencing SOX4 can effectively eliminate the drug resistance caused by lenvatinib treatment and inhibit the proliferation of cancer cells.Conclusions: This study highlights that SOX4 may serve as a promising therapeutic target for tumor treatment.

## 1. Introduction

Cancer ranks as a significant financial burden around the world. An estimated 19.3 million new cancer cases and almost 10.0 million cancer deaths occurred in 2020 worldwide [1]. In the United States, the cases of global cancer burden are estimated to reach 28.4 million in 2040, a 47% increase from 2020 [1,2]. In China, the age-standardized incidence rates (204.8 per 100,000) and age-standardized mortality rates (129.4 per 100,000) are higher than the global average level [3]. In recent years, along with the development of gene expression profiling and large-scale cancer multi-omics databases, comprehensive analysis is available not only to generate a global view of different tumor types but also to provide insight into their biological characteristics and molecular mechanisms. This is crucial to facilitate tumor diagnosis and potential drug screening to aid in the development of precision medicine strategies of cancer.

Sex-determining region Y-related (SRY) high-mobility group (HMG) box 4 (SOX4) is a member of the C subgroup of SRY-related HMG box transcription factor family. It is comprised of three domains. The HMG-box domain (59–138 amino acids) can directly change the chromatin architecture and, subsequently, alter gene expression [4]. The serine-rich region is a transactivation domain, whereas the glycine-rich region is a novel functional region [5]. SOX4 diversely regulates many cellular biological events such as embryonic development and cell fate determination [6]. SOX4 also plays an important role in tumorigenesis and cancer progression [7,8]. In addition, SOX4 is closely related to the multidrug resistance of lung cancer, colon cancer, breast cancer, leukemia, and other malignant tumors [9,10,11,12]. However, the specific drug resistance mechanism of SOX4 in LIHC is still unclear. These findings suggest SOX4 is a promising therapeutic target for cancer treatment.

In the present study, we firstly elucidated SOX4 expression and explored its correlation with prognosis in human tumor tissues. We also explored the genetic mutation and DNA promoter methylation level of SOX4 in different tumor types. Then, the correlation between the expression of SOX4 and immune cell infiltration, gene markers, and macrophage polarization across different tumor types, as well as immune score distribution, immune checkpoints-related gene, immunomodulator, immune, and molecular subtypes, were analyzed in liver hepatocellular carcinoma (LIHC). Moreover, we analyzed the association of SOX4 expression with DNA damage repair-, epithelial to mesenchymal transition (EMT)-, M6A methylation-, hypoxia-, ferroptosis-, and energy-metabolism-related genes in LIHC and constructed six prognostic nomograms to predict the 1-year, 3-year, and 5-year survival probabilities based on these SOX4-associated genes, respectively. Finally, the role of SOX4 in lenvatinib resistance and proliferation in hepatocellular carcinoma were identified in vitro.

## 2. Materials and Methods

### 2.1. Data Information

Transcriptome RNA-seq and clinical data for tumor tissues were downloaded from the Cancer Genome Atlas (TCGA) database (https://portal.gdc.cancer.gov/ (accessed on 13 July 2021)). The matched data for normal tissues were downloaded from the Genotype-Tissue Expression (GTEx) (https://gtexportal.org/home/ (accessed on 13 July 2021)). Tumor names and corresponding abbreviations are listed below: adrenocortical carcinoma (ACC), bladder urothelial carcinoma (BLCA), breast invasive carcinoma (BRCA), cervical squamous cell carcinoma and endocervical adenocarcinoma (CESC), cholangiocarcinoma (CHOL), colon adenocarcinoma (COAD), lymphoid neoplasm diffuse large B-cell lymphoma (DLBC), esophageal carcinoma (ESCA), glioblastoma multiforme (GBM), head and neck squamous cell carcinoma (HNSC), kidney chromophobe (KICH), kidney renal clear cell carcinoma (KIRC), kidney renal papillary cell carcinoma (KIRP), acute myeloid leukemia (LAML), brain lower grade glioma (LGG), liver hepatocellular carcinoma (LIHC), lung adenocarcinoma (LUAD), lung squamous cell carcinoma (LUSC), mesothelioma (MESO), ovarian serous cystadenocarcinoma (OV), pancreatic adenocarcinoma (PAAD), pheochromocytoma and paraganglioma (PCPG), prostate adenocarcinoma (PRAD), rectum adenocarcinoma (READ), sarcoma (SARC), skin cutaneous melanoma (SKCM), stomach adenocarcinoma (STAD), stomach and esophageal carcinoma (STES), testicular germ cell tumors (TGCT), thyroid carcinoma (THCA), thymoma (THYM), uterine corpus endometrial carcinoma (UCEC), uterine carcinosarcoma (UCS), and uveal melanoma (UVM).

### 2.2. Expression Analysis

TIMER2.0 (http://TIMER2.cistrome.org (accessed on 13 July 2021)) was used to study the expression of the SOX4 gene in tumor tissues and was matched to normal tissues. TIMER2.0 can analyze the tumor immunological, clinical, and genomic features across diverse cancer types. “Gene_DE” module of TIMER2.0 can identify the up-regulated genes or downregulated genes in the tumors. Considering the limited availability of normal samples in the TCGA database, Gene Expression Profiling Interactive Analysis 2 (GEPIA 2) (http://GEPIA.cancer-pku.cn (accessed on 13 July 2021)) was used to study the differential gene expression of SOX4 between 9 tumors and adjacent normal tissues, including DLBC, LAML, LGG, TGCT, THYM, UCS, ACC, OV, and SARC. GEPIA 2 can integrate the TCGA database and GTEx database. “Expression DIY-Box Plot” of GEPIA 2 can profile the tissue-wise expression of one gene or a multigene signature using a box plot. The parameters were as follows: |Log2FC| cutoff = 1, p-value cutoff = 0.01, log scale = “Yes”, jitter size = 0.04, and matched normal data = “Match TCGA normal and GTEx data”. “Expression DIY-Stage Plot” of GEPIA 2 can profile the expression of one gene in different tumor stages. The parameters were as follows: use major stage = “Yes”, and log scale = “Yes”. The UALCAN cancer database (http://ualcan.path.uab.edu (accessed on 13 July 2021)) was used to study the differential total-protein SOX4 expression between 6 tumors and adjacent normal tissues, including breast cancer, ovarian cancer, colon cancer, clear cell renal cell carcinoma, uterine corpus endometrial carcinoma, lung adenocarcinoma, and pediatric brain cancer. Moreover, GEPIA 2 was also used to analyze the expression and survival role of lncRNA in LIHC. The “CPTAC analysis” module of UALCAN was used to study the protein expression using data from the Clinical Proteomic Tumor Analysis Consortium (CPTAC) Confirmatory/Discovery dataset. The Human protein atlas (HPA) (https://www.proteinatlas.org/ (accessed on 13 July 2021)) was used to obtain immunohistochemistry images to map the protein expression levels of SOX4 in different tumor tissues.

### 2.3. Survival Analysis

GEPIA 2 and Kaplan–Meier Plotter (http://kmplot.com/analysis/ (accessed on 13 July 2021)) were used to study the correlation of SOX4 expression with patient overall survival (OS) and disease-free survival (DFS) across different tumor types, respectively. Survival Analysis-Survival Plots of GEPIA 2 can perform survival analysis and plot a Kaplan–Meier curve. The parameters were as follows: group cutoff = median, cutoff-high (%) = 50, cutoff-low (%) = 50, hazard ratio (HR) = “Yes”, 95% confidence interval = “Yes”, and axis units = “Months”. In this study, a “Kaplan-Meier plotter in pan-cancer” analysis was used to analyze the correlation between SOX4 expression and patient survival in 21 different cancers. The hazard ratio with 95% confidence intervals and log-rank *p*-value were also calculated and are shown in forest plots generated using GraphPad Prism 8.2.

### 2.4. Genetic Mutation Analysis

CBioPortal (https://www.cbioportal.org/ (accessed on 13 July 2021)) were used to elucidate the potential role of SOX4 mutation in data extracted from TCGA dataset. The mutation frequency of SOX4 gene was analyzed with the “Cancer Types Summary” module of cBioPortal. The types, sites, case number and 3D structure of SOX4 mutation were analyzed with the “mutations” module of cBioPortal. The correlation of SOX4 mutation with patient OS, DFS, disease-specific survival (DSF), and progression-free survival (PFS) were analyzed with the “Comparison/Survival” module of cBioPortal. The correlation of SOX4 mutation with the fraction of the copy number of the altered genome was analyzed using the “Plots” module of cBioPortal. In addition, we analyzed the correlation of SOX4 expression with the tumor mutation burden (TMB) and microsatellite instability (MSI) across all tumors of TCGA using Spearman’s correlation, and the correlation coefficient and *p*-value are shown in radar maps.

### 2.5. Methylation Analysis

The UALCAN cancer database was used to study the DNA promoter methylation level of SOX4 in tumor types. The “TCGA analysis” module of UALCAN was used to obtain the DNA promoter methylation level of SOX4. Especially, the correlation between the specific methylation site and SOX4 expression in LIHC was investigated with MEXPRESS (https://mexpress.be (accessed on 13 July 2021)). MEXPRESS can visualize the TCGA expression, DNA methylation, and clinical data, as well as the relationships among them.

### 2.6. ceRNA Regulatory Network Analysis

Six target prediction databases were used to predict the potential upstream binding miRNAs of SOX4, including DIANA (http://diana.imis.athena-innovation.gr/DianaTools/index.php (accessed on 13 July 2021)), PITA (https://genie.weizmann.ac.il/pubs/mir07/mir07_data.html (accessed on 13 July 2021)), TargetScan (http://www.targetscan.org/vert_71/ (accessed on 13 July 2021)), miRTarBase (https://mirtarbase.cuhk.edu.cn/ (accessed on 13 July 2021)), miRmap (https://mirmap.ezlab.org/ (accessed on 13 July 2021)), and mirDIP (http://ophid.utoronto.ca/mirDIP/ (accessed on 13 July 2021)). A Venn analysis (http://www.interactivenn.net/ (accessed on 13 July 2021)) was used to obtain and visualize the overlapped miRNA that appeared in at least four target prediction databases. The Encyclopedia of RNA Interactomes (ENCORI) (http://starbase.sysu.edu.cn/ (accessed on 13 July 2021)) was used to perform the miRNA expression analysis, survival analysis, and miRNA-target analysis in LIHC. ENCORI Pan-Cancer Analysis Platform can decode Pan-Cancer Networks of the lncRNAs, miRNAs, pseudogenes, snoRNAs, RNA-binding proteins, and all protein-coding genes by analyzing their expression profiles across 32 tumor types integrated from TCGA project. Next, LncBase Predicted v.2 (http://carolina.imis.athena-innovation.gr/diana_tools/web/ (accessed on 13 July 2021)) was used to predict the candidate lncRNAs of miRNA. ENCORI was also used to perform miRNA differential expression analysis, survival analysis, and miRNA-target analysis in LIHC.

### 2.7. Immune Characteristics Analysis

TIMER2.0 and TISIDB (http://cis.hku.hk/TISIDB/index.php (accessed on 13 July 2021)) were used to study the correlation of SOX4 expression with the immune characteristics of tumors. The “Gene” module of TIMER2.0 was used to study the correlation between SOX4 expression and the immune cells infiltration. The relationship between SOX4 expression and macrophage polarization was also studied. The “Gene_Corr” module of TIMER2.0 was used to study the correlation between SOX4 expression and the markers of immune infiltrating cells. In addition, the association between SOX4 expression and the subtypes immunomodulators was explored with the “Subtype” module of the TISIDB and the “Immunomodulator” module of the TISIDB in LIHC, respectively.

### 2.8. Prognostic Model Establishment

The raw counts of RNA-sequencing data (level 3) and corresponding clinical information of LIHC were obtained from the TCGA dataset. The least absolute shrinkage and selection operator (LASSO) regression technique was used for the predictor selection. Then, the multivariate analysis was used to identify the independent factors (two-sided *p*-value < 0.05) and generate a final nomograms. In addition, the LIHC patients were divided into two groups (high-risk and low-risk) based on the median risk score. The expression profiles of the prognostic genes in the two groups were visualized using a cluster heatmap. The predictive accuracy of the nomograms was measured with the area under receiver operating characteristic curve (AUC) of the receiver operating characteristic (ROC) curve.

### 2.9. Cell Culture

Human hepatocellular carcinoma cell lines HepG2 and Huh7 were purchased from Shanghai Cell Bank, Chinese Academy of Sciences. HepG2 cells were cultured in MEM medium containing 10% fetal bovine serum, supplemented with 2 mM glutamine, 100 U/mL penicillin and 100 μg/mL streptomycin. The culture medium of the Huh7 cells was DMEM containing 10% fetal bovine serum, and the final concentration was 100 U/mL penicillin and 100 μg/mL streptomycin. Both cells were cultured in a 5% CO_2_ incubator at 37 °C.

### 2.10. Protein Extraction and Western Blotting

Total protein was extracted from the HepG2 and Huh7 cells using RIPA lysate containing protease inhibitors (Solarbio, Beijing, China). The cells were collected with a scraper and lysed on ice for 30 min. Centrifugation was conducted at 4 °C, 12,000 rpm for 30 min. After centrifugation, the supernatant was discarded and the protein concentration was quantified using the BCA protein concentration assay kit (Solarbio, Beijing, China). Protein samples were separated with SDS-PAGE and transferred to PVDF membrane (Millipore, Billerica, MA, USA). The membrane was blocked with 5% skimmed milk powder at 37 °C for 1 h. The cells were incubated overnight with anti-SOX4 (Affinity, DF2610, 1:1000) antibody. Finally, ImageJ software (National Institutes of Health, version 1.8.0.345) was used to analyze and process the protein strength.

### 2.11. Cell Proliferative Assay

After cell counting, an appropriate amount of fresh medium was added to prepare a cell suspension with a cell concentration of approximately 5 × 104/mL cells. The cells were seeded in 96-well plates, and 100 μL cell suspension was inoculated into each well, approximately 5000 cells per well. The 96-well plates were placed in an incubator at 37 °C and 5% CO_2_. After the cells were adherent, the cells were treated with lenvatinib or SOX4 silencing combined with lenvatinib (6 wells in each group) for 72 h. The old medium in the 96-well plate was discarded and replaced with a mixed solution of fresh medium and CCK8 (medium:CCK8 was 10:1). After incubation in an incubator for 2 h, the OD value of each well sample was measured at a wavelength of 450 nm with a multifunctional microplate reader, and the cell survival rate was calculated.

### 2.12. Trypan Blue Staining

Cells were seeded in culture flasks and placed in an incubator at 37 °C, 5% CO_2_. After the cells were adherent, the cells were treated with lenvatinib or knockout of SOX4 combined with lenvatinib and continued to incubate for 48 h. Trypsin was added to digest cells and prepare a single cell suspension (10^6^/mL cells). The cell suspension was mixed with 0.4% trypan blue solution at a ratio of 9:1. After three minutes, the living cells and dead cells were counted using a counting plate.

### 2.13. Detection of Apoptosis and Necrosis

Trypsin was added to the tumor cells treated with lenvatinib or SOX4 knockdown. After being centrifuged at 4 °C 1000 rpm for 5 min, the supernatant was discarded. The cells were resuspended in 1 mL of pre-cooled PBS, centrifuged at 1000 rpm at 4 °C for 5 min, and the supernatant was discarded. The cells were resuspended with 1 mL Cell Stain Buffer, followed by 5 μL Hoechst 33342 Stain and 5 μL PI Stain. Finally, the fluorescence intensity was detected using a fluorescence microscope after smear.

### 2.14. Statistical Analysis

R software version 4.0.0 and SPSS version 24.0 were used for the statistical analyses. The data are expressed as the means ± SD. The statistical analysis was performed using one-way ANOVA. A two-sided *p*-value < 0.05 was considered to be statistically significant.

## 3. Results

### 3.1. Expression of SOX4 across Tumor Types

**Figure 1:**
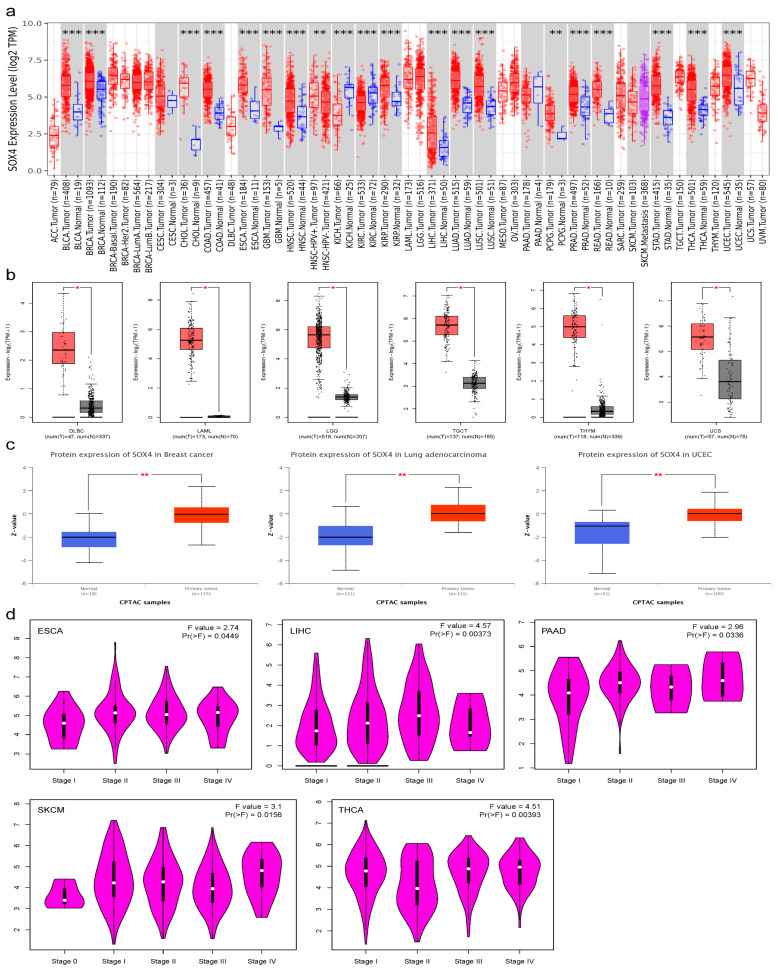
Expression of SOX4 across tumor types: (**a**) expression of SOX4 gene in different tumors or specific tumor subtypes determined using TIMER2; (**b**) expression of SOX4 gene in different tumors determined using GEPIA 2; (**c**) protein expression of SOX4 gene in different tumors determined using UALCAN; (**d**) correlation between SOX4 expression and the different pathological stages of tumors determined using GEPIA2. * *p* < 0.05, ** *p* < 0.01, and *** *p* < 0.001.

We first compared the SOX4 mRNA expression based on the TCGA data via the TIMER tool. As shown in Figure 1a, compared with the adjacent normal tissues, the expression of SOX4 was markedly increased in 18 tumor types or specific cancer subtypes including BLCA, BRCA, CHOL, COAD, ESCA, GBM, HNSC-HPV+, HNSC, KIRP, LIHC, LUAD, LUSC, PCPG, PRAD, READ, STAD, THCA, and UCEC, but it was significantly decreased in KICH and KIRC. There was no statistically significant difference in CESC, PAAD, and SKCM. Considering the limited availability of normal data in the TCGA database, we integrated the TCGA and GTEx data to study the difference in SOX4 mRNA expression in nine tumor types. As shown in Figure 1b, compared with the adjacent normal tissues, SOX4 expression was markedly increased in DLBC, LAML, LGG, TGCT, THYM, and UCS. However, there was no statistically significant difference in ACC, OV, and SARC. Moreover, we performed a protein expression analysis of SOX4 for breast cancer, colon cancer, ovarian cancer, clear cell renal cell carcinoma, UCEC, LUAD, and pediatric brain cancer using the “CPTAC analysis” module of UALCAN. As shown in Figure 1c, compared with the adjacent normal tissues, the protein expression of SOX4 was markedly increased in breast cancer, LUAD, and UCEC. There is no information available for SOX4 in other tumor types. The immunohistochemical staining of SOX4 in various tumors are shown in Supplementary Materials Figure S1. We also evaluated the correlation between the expression of SOX4 and the tumors pathological stages. SOX4 expression was found to significantly correlate with the pathological stages of ESCA, LIHC, PAAD, SKCM, and THCA (Figure 1d).

### 3.2. Prognostic Values of SOX4 across Tumor Types

**Figure 2:**
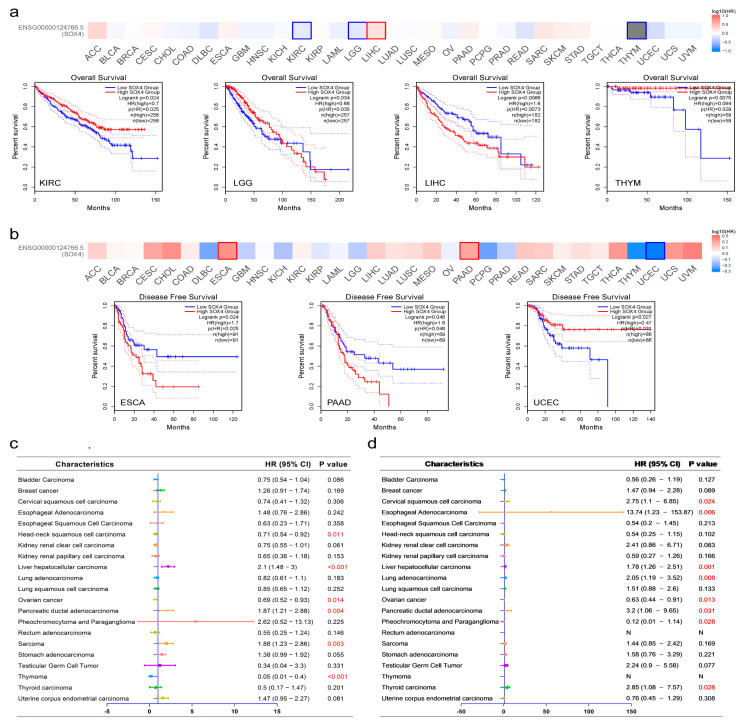
Prognostic values of SOX4 across tumor types: (**a**) correlation of SOX4 expression with OS of different tumors in the TCGA determined using GEPIA2; (**b**) correlation of SOX4 expression with DFS of different tumors in the TCGA determined using GEPIA2; (**c**) forest plot of the correlation of SOX4 expression with OS across 21 tumor types determined using Kaplan–Meier Plotter; (**d**) forest plot of the correlation of SOX4 expression with DFS across 21 tumor types determined by Kaplan–Meier Plotter.

We studied the prognostic values of SOX4 in patients. According to the GEPIA2 analysis results, high SOX4 expression was significantly correlated with poor OS in LGG and LIHC, and low SOX4 expression was significantly associated with poor OS in KIRC and THYM (Figure 2a). Moreover, high SOX4 expression was significantly correlated with poor DFS in ESCA and PAAD, and low SOX4 expression was significantly associated with poor DFS in UCEC (Figure 2b). According to the Kaplan–Meier analysis results, high SOX4 expression was significantly correlated with poor OS in LIHC, pancreatic ductal adenocarcinoma, and SARC. However, low SOX4 expression was significantly associated with poor OS in HNSC, ovarian cancer, and THYM (Supplementary Materials Figure S2a). For DFS, high SOX4 expression was significantly correlated with poor DFS in cervical squamous cell carcinoma, esophageal adenocarcinoma, LIHC, lung adenocarcinoma, pancreatic ductal adenocarcinoma, and THCA. However, low SOX4 expression was significantly associated with poor DFS in ovarian cancer and PCPG (Supplementary Materials Figure S2b). As shown in Figure 2c,d, the correlations of SOX4 expression with the OS and DFS of patients across 21 tumor types based on the Kaplan–Meier Plotter are exhibited in forest plots, respectively.

### 3.3. Genetic Mutation of SOX4 across Tumor Types

**Figure 3:**
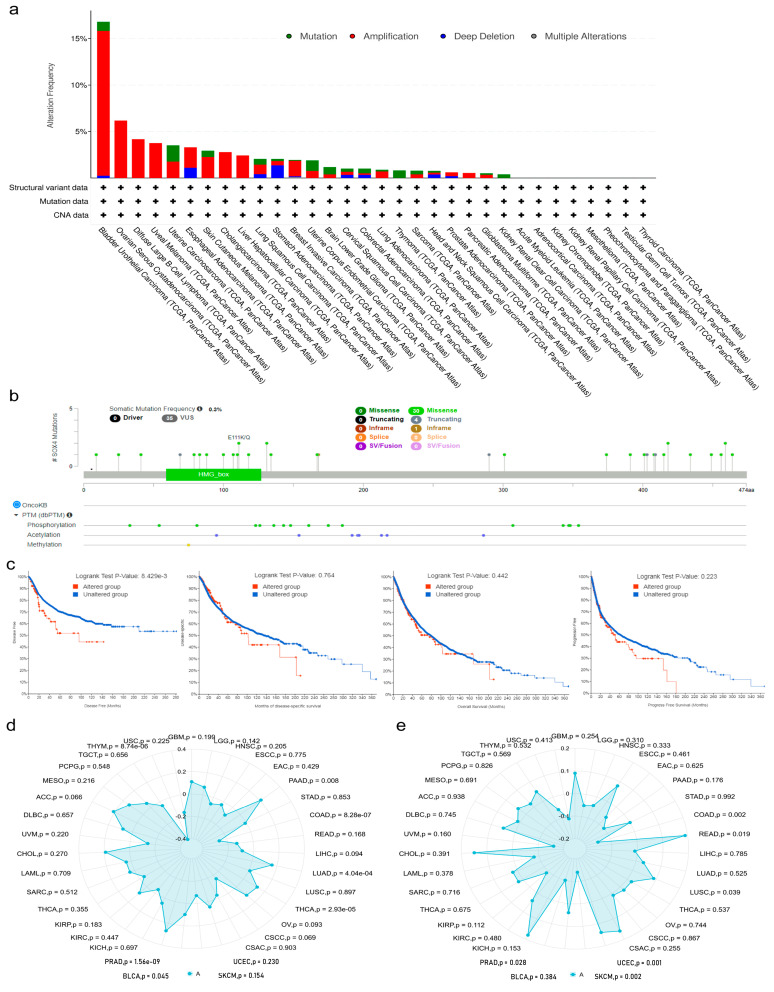
Genetic mutation of SOX4 across tumor types: (**a**) genetic mutation frequency of SOX4 in different tumors; (**b**) genetic mutation types, sites, and case number of SOX4 in different tumors; (**c**) correlation of SOX4 expression with survival prognosis (DFS, PFS, OS, and DSS); (**d**) radar chart of the correlation of SOX4 expression with TMB; (**e**) radar chart of the correlation of SOX4 expression with MSI.

Genetic mutation is a major issue that contributes to tumorigenesis and prognosis. To elucidate the potential role of SOX4 mutation in tumors, we checked the mutation frequency of SOX4 gene across 32 human tumors using TCGA data from the cBioPortal database. As shown in Figure 3a, 24 of the 32 analyzed tumors had more than one mutation, and 8 tumors showed no mutation, including LAML, ACC, KICH, KIRP, MESO, PCPG, TGCT, and THCA. The patients with BLCA had the highest mutation frequency of the SOX4 gene (16.79%), followed by OV (6.16%). Among all types of genetic mutations, the “amplification” type was the most frequent alteration. Notably, the “amplification” type was the sole alteration of the SOX4 gene in the patients with CHOL, DLBC, LIHC, OV, PAAD, and UVM. In addition, the “mutated” type was the sole alteration of the SOX4 gene in the patients with KIRC and THYM. The types, sites, and case number of the SOX4 mutation (including missense, truncating, and in-frame) are presented in Figure 3b. The frequency of the somatic mutation was 0.3%. There were 35 mutations sites between 0 and 474 amino acids. Missense was the main type of genetic mutation. As shown in Figure 3c, compared with the samples without any alteration of the SOX4 gene (i.e., unaltered group, number of cases = 5277), the samples with at least one alteration of the SOX4 (i.e., altered group, number of cases = 106) showed poorer prognosis with regard to DFS but not PFS, OS, and DSS. As shown in Supplementary Materials Figure S3, the mutation count of the SOX4 gene was positively correlated with the fraction of the copy number altered genome (Spearman = 0.34, Pearson = 0.21). Meanwhile, we studied the correlation of SOX4 expression with TMB and MSI. The results indicate that the aberrant expression of SOX4 was positively associated with TMB in the patients with BLCA, LUAD, PAAD, PRAD, and THCA, but it was negatively correlated with that of COAD and THYM (Figure 3d, Supplementary Materials Figure S4). The aberrant expression of SOX4 was positively associated with MSI in the patients with LUSC, READ, and UCEC and negatively correlated with that of COAD, PRAD, and SKCM (Figure 3e, Supplementary Materials Figure S5).

### 3.4. ceRNA Regulatory Network of SOX4 in LIHC

**Figure 4:**
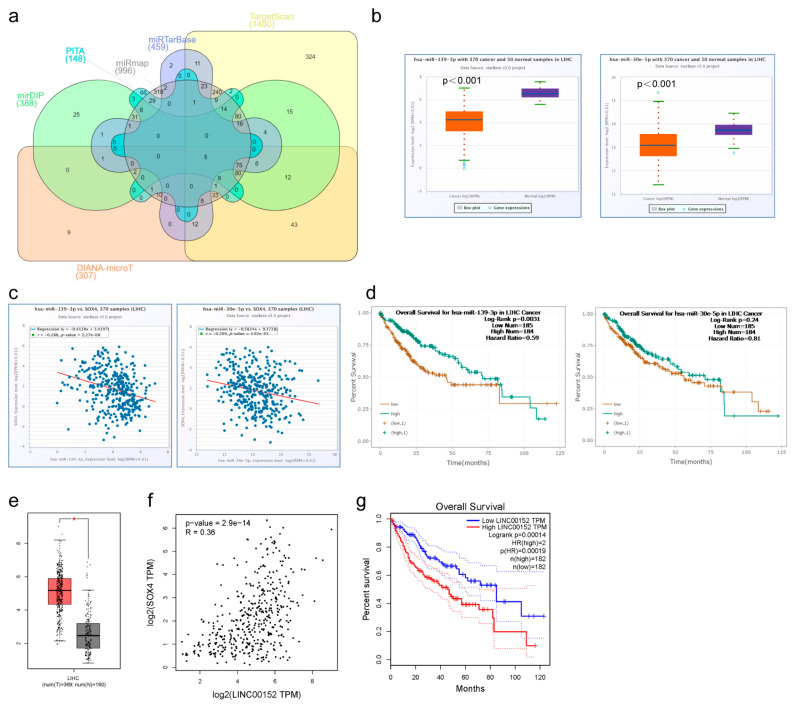
ceRNA regulatory network of SOX4 in LIHC: (**a**) predicted miRNA of SOX4 using six target prediction databases shown in the Venn diagram; (**b**) expression of hsa-miR-139-3p and hsa-miR-30e-5p; (**c**) correlation of SOX4 expression with hsa-miR-139-3p and hsa-miR-30e-5p; (**d**) correlation of hsa-miR-139-3p and hsa-miR-30e-5p expression with OS; (**e**) expression of LINC00152; (**f**) correlation of SOX4 expression with LINC00152; (**g**) correlation of LINC00152 expression with OS. * *p* < 0.05.

Increasing evidence shows that changes to and dysfunction of lncRNA lead to abnormal gene expression and promote the formation, progression, and metastasis of many types of cancer. lncRNAs can absorb miRNA and then promote mRNA expression. In order to explore the relationship between SOX4 and lncrna and miRNA we, therefore, analyzed the ceRNA regulatory network of SOX4 in tumor tissues of LIHC. We firstly predicted the potential miRNA of SOX4 with six target prediction databases, including DIANA, PITA, TargetScan, miRTarBase, miRmap, and mirDIP. As shown in the Venn diagram (Figure 4a), there are 215 overlapping miRNAs identified in at least four of the six target prediction databases. Among these miRNAs, only two miRNAs (hsa-miR-139-3p and hsa-miR-30e-5p) were significantly lower than that of the adjacent normal tissues and negatively correlated with the expression of SOX4 in LIHC (Figure 4b,c). Moreover, has-miR-139’3p’s expression was significantly correlated with poor OS in LIHC. However, there was no statistically significant difference between the expression of hsa-miR-30e-5p and OS in LIHC (Figure 4d). Therefore, we further focused on the role of hsa-miR-139-3p in LIHC and predicted its upstream lncRNAs. There were 476 lncRNAs identified using LncBase Predicted v.2 database. Among these lncRNAs, only LINC00152’s expression was significantly higher than that of the adjacent normal tissue and was positively correlated with the expression of SOX4 in LIHC (Figure 4e,f). Moreover, LINC00152’s expression was correlated with poor OS in LIHC (Figure 4g). LINC00152’s expression was also negatively correlated with the expression of hsa-miR-139-3p in LIHC. So far, we found that LINC00152 could function as a ceRNA to regulate SOX4 expression by sponging hsa-miR-139-3p in LIHC.

### 3.5. Immune Characteristics of SOX4 in LIHC

**Figure 5:**
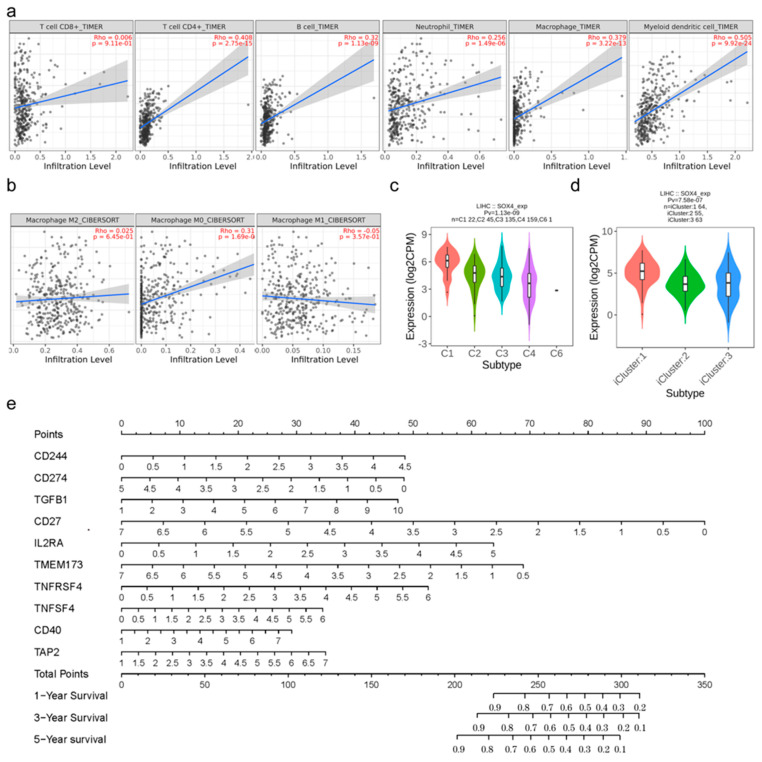
Immune characteristics of SOX4 in LIHC: (**a**) correlation of SOX4 expression with immune cell infiltration; (**b**) correlation of SOX4 expression with macrophage polarization; (**c**) correlation of SOX4 expression with immune subtype; (**d**) correlation of SOX4 expression with molecular subtype; (**e**) nomogram constructed based on ten SOX4-associated immune-related genes to predict LIHC prognosis.

The tumor immune microenvironment (TIME) is involved in tumor clonal evolution, growth, metastasis, prognosis, and drug resistance, as well as therapeutic outcome. Therefore, we further analyzed the immune characteristics of SOX4 in LIHC. First, we assessed the relationship between SOX4 expression and the infiltration level of immune cells. The correlations between SOX4 expression and the immune infiltrating cells (including CD8+ T cells, CD4+ T cells, B cells, neutrophils, macrophages, and myeloid dendritic cells) across various tumor types were visualized using cluster heatmaps (Supplementary Materials Figure S6). Especially in LIHC, the result shows that SOX4 expression was significantly correlated with tumor purity in TIMER. Notably, SOX4 expression was positively correlated with CD4+ T cells, B cells, neutrophils, macrophages, and myeloid dendritic cells but not CD8+ T cells (Figure 5a). For the macrophage polarization analysis, SOX4 expression was positively correlated with M0 polarization but not with M1 or M2 polarization (Figure 5b). Second, we analyzed the relationship between SOX4 expression and the markers of CD8+ T cells, B cells, neutrophils, macrophages, myeloid dendritic cells, tumor-associated macrophages, monocytes, and natural killer cells, as well as Tfh, Th1, Th2, Th9, Th17, Th22, Treg, and exhausted T cells. The correlations between SOX4 expression and the markers of the above cells across various tumor types were visualized using cluster heatmaps (Supplementary Materials Figure S7). Especially in LIHC, the expression of SOX4 was positively associated with CD19, CD38, CD8A, CD8B, CXCR5, ICOS, BCL6, CCR3, GATA3, TGFBR2, IRF4, IL21R, IL23R, STAT3, CCR10, AHR, CCR8, CTLA4, CD68, CD80, CD86, XCL1, CD7, MPO, and CD1C and negatively associated with ARG1 and CD14.

Then, we analyzed the associations between SOX4 expression and the immune subtype, molecular subtype, and immunomodulators in LIHC. The associations between SOX4 expression and immune subtypes across human cancers are shown in Supplementary Materials Figure S8a. The association between SOX4 expression and molecular subtypes are shown in Supplementary Materials Figure S8b. Especially, the immune subtype analysis showed that the expressions of SOX4 were different in the C1, C2, C3, C4, C5, and C6 immune subtypes in LIHC (Figure 5c). Analogously, the molecular subtype analysis indicated that the expression of SOX4 was different in iCluster 1, iCluster 2, and iCluster 3 in LIHC (Figure 5d). The relationships between three kinds of immunomodulators (immunoinhibitor, immunostimulator, and MHC molecule) and SOX4 expression across human tumors are shown in Supplementary Materials Figure S9a–c. Especially in LIHC, Spearman’s analysis revealed that the expression of SOX4 was inversely correlated with the expression of all immunoinhibitors, including BTLA, CD96, CD244, CD274, CSF1R, CTLA4, HAVCR2, LAG3, LGALS9, PDCD1LG2, TGFB1, TGFBR1, and TIGIT (Supplementary Materials Figure S10). The expression of SOX4 was also found to positively correlate with the expression of 23 immunostimulators, including C10orf54, CD27, CD48, CD80, CD86, CD276, CXCL12, CXCR4, ENTPD1, HHLA2, ICOS, IL2RA, IL6R, TMEM173, TNFRSF4, TNFRSF8, TNFRSF9, TNFRSF18, TNFSF4, TNFSF9, TNFSF13B, TNFSF15, and ULBP1, and inversely correlate with the expression of 4 immunostimulators, including CD40, ICOSLG, LTA, and PVR (Supplementary Materials Figure S11). SOX4 expression was positively correlated with the expression of four MHC molecules, including DMB, DOA, DQA1, and DQA2, and was inversely correlated with the expression of six MHC molecules, including B2M, HLA-B, HLA-C, HLA-E, HLA-F, and TAP2 (Supplementary Materials Figure S12). Based on the above immunomodulators and SOX4, we further established a multigene prognostic model to predict LIHC prognosis. As shown in Supplementary Materials Figure S13a,b, 22 genes had nonzero coefficients in the LASSO analysis. A multivariate logistic regression indicated that CD244, CD274, TGFB1, CD27, IL2RA, TMEM173, TNFRSF4, TNFSF4, CD40, and TAP2 were independent risk factors for OS (Table 1). These ten independent genes were used to construct the nomogram (Figure 5e), in which the risk score = (0.4468) × CD244 + (−0.3822) × CD274 + (0.2257) × TGFB1 + (−0.6075) × CD27 + (0.5307) × IL2RA + (−0.4436) × TMEM173 + (0.3822) × TNFRSF4 + (0.248) × TNFSF4 + (0.1849) × CD40 + (0.2379) × TAP2. The LIHC patients were divided into two groups: high-risk and low-risk groups (Supplementary Materials Figure S13c). The survival time and survival status of the patients in the high-risk and low-risk groups are shown in Supplementary Materials Figure S13d. The expression profiles of the prognostic genes in the two groups were visualized using a cluster heatmap (Supplementary Materials Figure S13e). The survival analysis demonstrated that the high-risk group was associated with worse survival in LIHC (Supplementary Materials Figure S13f). Additionally, ROC curve analyses indicated that the model was reliable, and the AUCs for 1-year survival, 3-year survival, and 5-year survival were 0.77, 0.741, and 0.761, respectively (Supplementary Materials Figure S13g).

### 3.6. Prognostic Models Based on SOX4-Associated Genes in LIHC

**Figure 6:**
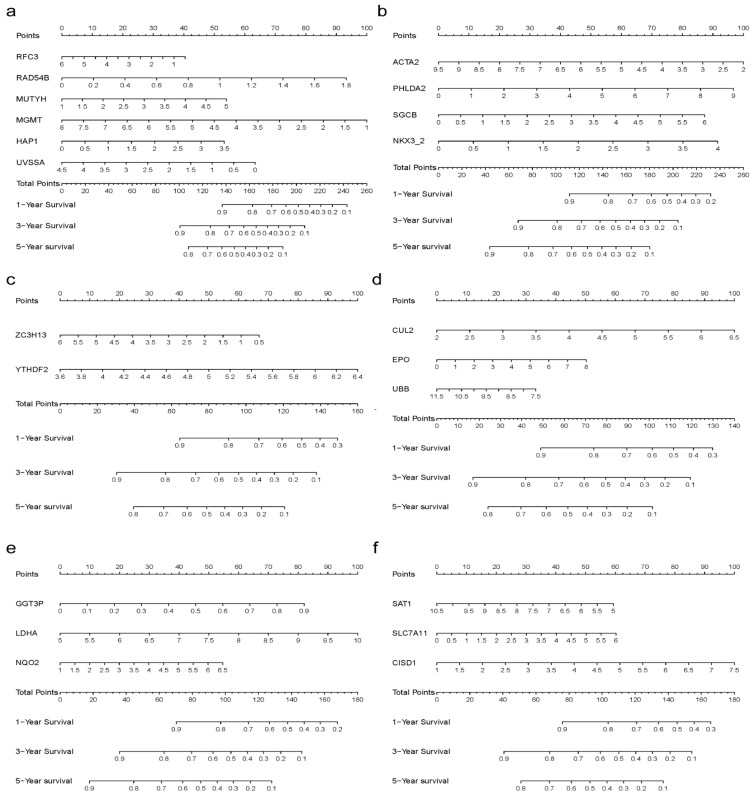
Prognostic models based on SOX4-associated genes in LIHC: (**a**) nomogram constructed based on six SOX4-associated DNA damage repair-related genes to predict LIHC prognosis; (**b**) nomogram constructed based on four SOX4-associated EMT-related genes to predict LIHC prognosis; (**c**) nomogram constructed based on two SOX4-associated M6A methylation-related genes to predict LIHC prognosis; (**d**) nomogram constructed based on three SOX4-associated hypoxia-related genes to predict LIHC prognosis; (**e**) nomogram constructed based on three SOX4-associated energy-metabolism-related genes to predict LIHC prognosis; (**f**) nomogram constructed based on three SOX4-associated ferroptosis-related genes to predict LIHC prognosis.

We further established six multigene prognostic models to predict LIHC prognosis based on SOX4-associated genes, respectively. The genes were initially collected through a comprehensive literature search. For the prognostic model based on the SOX4-associated DNA damage repair-related genes, 200 genes were collected and their correlations with SOX4 expression across various tumor types were visualized using cluster heatmaps (Supplementary Materials Figure S14a). In LIHC, 21 genes had nonzero coefficients in the LASSO analysis (Supplementary Materials Figure S14b,c). Multivariate logistic regression indicated that RFC3, RAD54B, MUTYH, MGMT, HAP, and UVSSA were independent risk factors for OS (Table 1). These genes were used to construct the nomogram (Figure 6a), where risk score = (−0.1752) × RFC3 + (1.4157) × RAD54B + (0.3664) × MUTYH + (−0.3797) × MGMT + (0.406) × HAP1 + (−0.3656) × UVSSA. The risk scores of the high-risk group and low-risk group are shown in Supplementary Materials Figure S14d. The survival time and survival status of the patients are shown in Supplementary Materials Figure S14e. The expression profiles of the prognostic genes are visualized in Supplementary Materials Figure S14f. The Kaplan–Meier survival analysis is shown in Supplementary Materials Figure S14g. The ROC curve analyses are shown in Supplementary Materials Figure S14h.

For the prognostic model based on the SOX4-associated EMT-related genes, 95 genes were collected and their correlations with SOX4 expression across various tumor types were visualized using cluster heatmaps (Supplementary Materials Figure S15a). In LIHC, nine genes had nonzero coefficients in the LASSO analysis (Supplementary Materials Figure S15b,c). A multivariate logistic regression indicated that ACTA2, PHLDA2, SGCB, and NKX3-2 were independent risk factors for OS (Table 1). These genes were used to construct the nomogram (Figure 6b), in which the risk score = (−0.2898) × ACTA2 + (0.2364) × PHLDA2 + (0.3189) × SGCB + (0.5039) × NKX3-2. The risk scores, survival time and survival status, expression profiles, Kaplan–Meier survival analysis, and ROC curve analyses are shown in Supplementary Materials Figure S15d–h. A multivariate logistic regression indicated that ZC3H13 and YTHDF2 were independent risk factors for OS (Table 1). These genes were used to construct the nomogram (Figure 6c), in which risk score = (−0.3353) × ZC3H13 + (1.0005) × YTHDF2.

For the prognostic model based on the SOX4-associated hypoxia-related genes, 75 genes were collected and their correlations with SOX4 expression across various tumor types were visualized using cluster heatmaps (Supplementary Materials Figure S16a). In LIHC, 12 genes had nonzero coefficients in the LASSO analysis (Supplementary Materials Figure S16b,c). A multivariate logistic regression indicated that CUL2, EPO, and UBB were independent risk factors for OS (Table 1). These genes were used to construct the nomogram (Figure 6d), in which risk score = (0.6505) × CUL2 + (0.1865) × EPO + (−0.2321) × UBB. The risk scores, survival time and survival status, expression profiles, Kaplan–Meier survival analysis, and ROC curve analyses are shown in Supplementary Materials Figure S16d–h.

For the prognostic model based on the SOX4-associated energy-metabolism-related genes, 100 genes were collected and their correlations with SOX4 expression across various tumor types were visualized using cluster heatmaps (Supplementary Materials Figure S17a). In LIHC, 10 genes had nonzero coefficients in the LASSO analysis (Supplementary Materials Figure S17b,c). A multivariate logistic regression indicated that GGT3P, LDHA, and NQO2 were independent risk factors for OS (Table 1). These genes were used to construct the nomogram (Figure 6e), in which risk score = (2.395) × GGT3P + (0.5369) × LDHA + (0.2521) × NQO2. The risk scores, survival time and survival status, expression profiles, Kaplan–Meier survival analysis, and ROC curve analyses are shown in Supplementary Materials Figure S17d–h.

For the prognostic model based on the SOX4-associated ferroptosis-related genes, 24 genes were collected and their correlations with SOX4 expression across various tumor types were visualized using cluster heatmaps (Supplementary Materials Figure S18a). In LIHC, nine genes had nonzero coefficients in the LASSO analysis (Supplementary Materials Figure S18b,c). Multivariate logistic regression indicated that SAT1, SLC7A11, and CISD1 were independent risk factors for OS (Table 1). These genes were used to construct the nomogram (Figure 6f), in which risk score = (−0.2718) × SAT1 + (0.2648) × SLC7A11 + (0.4011) × CISD1. The risk scores, survival time and survival status, expression profiles, Kaplan–Meier survival analysis, and ROC curve analyses are shown in Supplementary Materials Figure S18d–h.

### 3.7. Role of SOX4 Knockdown in Lenvatinib-Treated LIHC Cells

**Figure 7:**
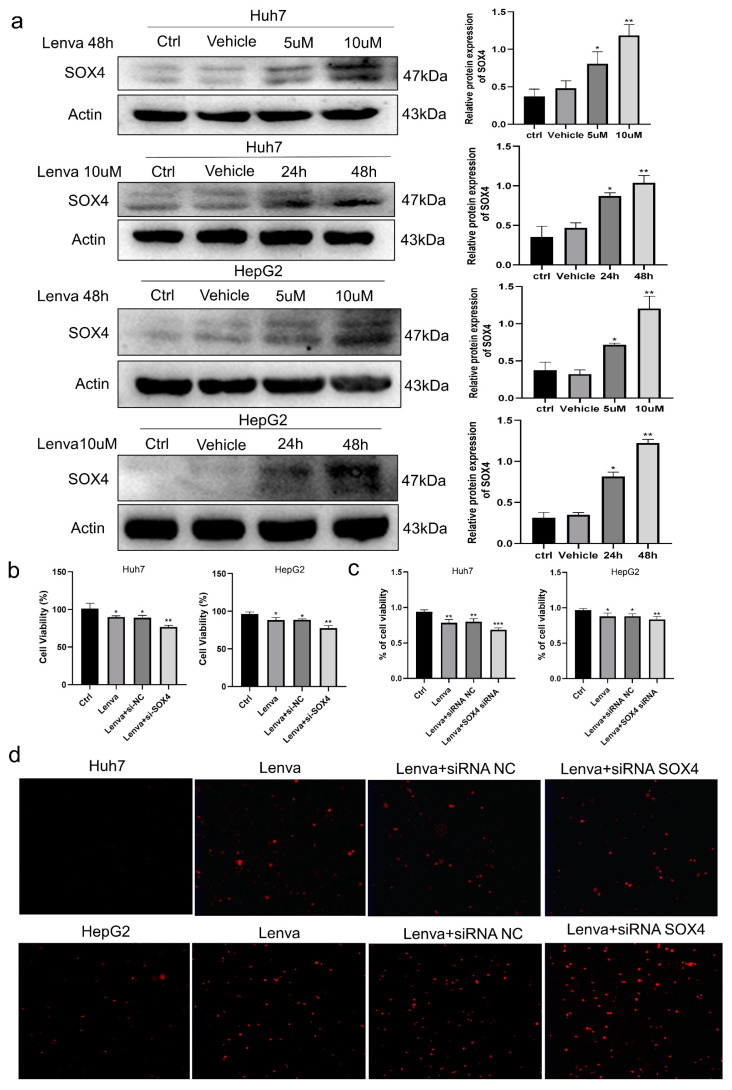
SOX4 silencing increased the sensitivity of lenvatinib to hepatocellular carcinoma cells and cell necrosis: (**a**) Western blotting of SOX4 in Huh7 or HepG2 cells treated with 0, 5, and 10 uM lenvatinib for 24 or 48 h; (**b**) cell viability of Huh7 and HepG2 cells treated with 10 uM lenvatinib and lenvatinib combined with knockdown of SOX4 was detected; (**c**) trypan blue staining of Huh7 and HepG2 cells treated with lenvatinib combined with knockdown of SOX4; (**d**) apoptosis and necrosis staining of Huh7 and HepG2 cells after treatment with lenvatinib combined with knockdown of SOX4 for 48 h. * *p* < 0.05, ** *p* < 0.01, *** *p* < 0.001.

To further validate the results of SOX4 in above pan-cancer analysis and LIHC, we analyzed the role of SOX4 knockdown in lenvatinib-treated LIHC cells in vitro. As known, lenvatinib was approved as a first-line drug for the treatment of unresectable hepatocellular carcinoma [13]. Firstly, Huh7 and HepG2 cells were treated with 0, 5, or 10 uM lenvatinib for 24 or 48 h, respectively. The results show that lenvatinib increased the expression of SOX4 in HepG2 and Huh7 cells in a time- and dose-dependent manner (Figure 7a). In order to further explore the relationship between SOX4 expression and lenvatinib resistance, we knocked down SOX4 in LIHC cells. HepG2 or Huh7 cells treated with lenvatinib (10 uM) were treated with SOX4 siRNA for 48 h to analyze cell proliferation and necrosis. The CCK8 assay and trypan blue staining showed that, compared with the lenvatinib treatment group, lenvatinib combined with SOX4 silencing significantly inhibited the proliferation and viability of HepG2 and Huh7 cells (Figure 7b,c). The apoptosis and necrosis experiments also showed that, compared with lenvatinib treatment alone, lenvatinib combined with SOX4 silencing significantly increased the necrosis of HepG2 and Huh7 cells (Figure 7d).

## 4. Discussion

A comprehensive analysis is of great importance for the accurate diagnosis and effective therapy of tumors [14,15]. This is crucial not only to tackle the biological characteristics and molecular mechanisms of different cancer types but also to provide some insight for researchers into the development of more precise therapeutic strategies against various cancers. Therefore, many researchers are focusing on exploring ways to generate a global view of different cancer types [16,17]. The present study aimed to visualize the expression, survival, mutations, methylation, ceRNA network, immunity, and prognostic models, as well as explore the potential role, of SOX4 across different tumor types and specific LIHC using different online tools.

SOX4 is a critical transcription factor that is involved in many cellular events, such as stemness, differentiation, progenitor development, and tumorigenesis, contributing to diverse pathological conditions [4,18]. Although emerging research has evaluated the expression level and function of SOX4 in certain tumors, its roles across various tumor types, especially aspects related to prognostic potential and clinical significance, have not been systematically studied. In this study, we comprehensively analyzed the expression levels of SOX4 between tumors and normal tissues for the first time. Our study indicated that the mRNA expression of SOX4 was upregulated in most of the tumors. In addition, the study indicated that the protein expression of SOX4 was significantly higher in breast cancer, LUAD, and UCEC. It was also found that the expression of SOX4 was correlated with the pathological stages of ESCA, LIHC, PAAD, SKCM, and THCA. Overall, these results strongly suggest that SOX4 might exert specific and even contrasting functions in different tumor types.

To further clarify the functions of SOX4 in different tumor types, this study analyzed the correlation between SOX4 expression and patients’ prognosis. Our study shows that SOX4 upregulation was associated with a poor prognostic outcome in several tumors, especially in LIHC. Therefore, SOX4 might be a potential prognostic and diagnostic marker of survival outcomes. Indeed, the prognostic values of SOX4 have previously been reported in some malignant tumors. Some experimental studies have revealed that SXO4 expression is correlated with poor prognosis in patients with tumors, such as CHOL [19] and LAML [20]. The prognostic value of SOX4 was partly validated again in our study, despite some controversial results. The role of SOX4 in these cancers is still open to question and further discussion.

To clarify the underlying mechanisms that drive the observed results, we further checked the genetic mutations and DNA promoter methylation level of SOX4. At the gene level, we found that the samples with at least one alteration of the SOX4 gene showed poor prognosis. In addition, the expression of SOX4 was significantly associated with MSI and TMB status. At the DNA level, the promoter methylation level of SOX4 was upregulated in ESCA, KIRC, KIRP, LIHC, LUSC, PAAD, SARC, and TGCT, while it was downregulated in BLCA, BRCA, HNSC, THCA, and UCEC. Especially in LIHC, a strong correlation between SOX4 expression and the methylation sites was observed. Indeed, the accumulation of genetic mutations and epigenetic alterations play a pivotal role during tumorigenesis. It is well known that many tumors harbor several genetic mutations. Genetic mutations drive the development of tumors and affect tumor prognosis [21]. Epigenetic alterations regulate gene expression and establish cell-type-specific temporal and spatial expression patterns [22]. These may partially explain the complex roles of SOX4 in the prognosis of various tumors.

ceRNA regulatory networks are recognized as important regulators of gene expression at the posttranscriptional level. Accumulating evidence indicates that ceRNA regulatory networks regulate many biological processes, especially in tumors [23]. In our study, we firstly predicted the potential miRNAs of SOX4. We found that hsa-miR-139-3p was not only significantly decreased but also negatively correlated with SOX4 expression and poor OS in LIHC. The previously reported miR-363-3P and miR-129-2 were also found to be downregulated in HCC and negatively regulate SOX4 levels in vitro [24,25]. Indeed, hsa-miR-139-3p functions as a biomarker in different cancer types, such as colorectal cancer and hepatocellular carcinoma [26]. We further predicted the upstream lncRNAs of hsa-miR-139-3p. We found that LINC00152 was not only significantly higher than that of the adjacent normal tissues but also positively correlated with SOX4 expression and poor OS in LIHC. Accumulated evidence shows that LINC00152 plays an important role in carcinogenesis by disturbing various signaling pathways in several cancers [27]. Nevertheless, no previous study has evaluated the importance of the role of the LINC00152/hsa-miR-139-3p/SOX4 ceRNA regulatory network in LIHC. In our study, we evaluated the prognostic values of LINC00152/hsa-miR-139-3p/SOX4, which will provide novel insight into the treatment of LIHC.

The TIME was another crucial aspect of this study. The TIME acted as a crucial regulator in the development, progression, and immune escape of various types of cancer [28]. A better definition and understanding of the TME could open novel avenues for the curative treatment of metastatic tumors. In this study, the analysis revealed that SOX4 expression was associated with CD4+ T cells, B cells, neutrophils, macrophages, and myeloid dendritic cells but not CD8+ T cells. Moreover, SOX4 expression was correlated with the M0 polarization of macrophages. We also confirmed the relationship between SOX4 expression and the markers of immune-infiltrating cells, especially in LIHC. We further investigated the associations between SOX4 expression and the immune subtypes, molecular subtypes, and immunomodulators in LIHC. We further established a multigene prognostic model to predict the clinical prognosis of LIHC. Notably, the model also displayed excellent predictive discrimination. Thus, this model is a sensitive prediction tool under the promise of guaranteeing accuracy.

It is well known that tumors are shaped by combined action of lifestyle, environmental, eating habits, physical activity, and genetic factors. Some reliable models that can predict the prognosis are urgently required. As known, aberrant DNA damage, EMT, M6A methylation, hypoxia, energy metabolism, and ferroptosis are important events generally involved in tumor onset and development [29,30,31,32,33,34]. Therefore, we established six multigene prognostic models to predict LIHC prognosis based on the SOX4-associated genes, including DNA damage repair-related genes, EMT-related genes, M6A methylation-related genes, hypoxia-related genes, energy-metabolism-related genes, and ferroptosis-related genes. The estimated 1-, 3-, and 5-year survival probabilities could easily be calculated using the nomograms. All models also displayed excellent predictive discrimination with regard to the AUCs for the 1-, 3-, and 5-year survival. To date, there exists no study that has evaluated the prognostic values of these genes in patients with LIHC. The present study might be the first to have constructed nomograms for the prediction of LIHC prognosis based on the systematic assessment of cellular core genes.

Lenvatinib is a multitarget tyrosine kinase inhibitor that inhibits the growth and angiogenesis of malignant cells by inhibiting the activation of multiple receptor tyrosine kinase signaling pathways, thereby effectively treating a variety of cancers. In 2018, lenvatinib was approved for first-line treatment in patients with advanced hepatocellular carcinoma in the United States, the European Union, Japan, and China [35]. However, some patients developed drug resistance after using lenvatinib for a period of time. Increasing evidence shows that SOX4 is closely related to tumor drug resistance. In our study, we found that lenvatinib treatment upregulated the expression of SOX4 in Huh7 and HepG2 cells in a dose- and time-dependent manner, indicating that SOX4 may associated with lenvatinib drug resistance in LIHC. In addition, we also demonstrated that, compared with lenvatinib treatment, lenvatinib treatment combined with SOX4 silencing significantly inhibited the proliferation of LIHC cells and increased necrosis.

## 5. Conclusions

In conclusion, SOX4 was widely overexpressed in tumor tissues and associated with unfavorable prognoses, genetic mutation, and DNA methylation level, especially in LIHC. Moreover, the results provide novel evidence supporting LINC00152/hsa-miR-139-3p/SOX4 as a crucial target for the treatment of LIHC. The results also provide insight into the significant role of SOX4 expression in immune cell infiltration, macrophage polarization, immune subtype, molecular subtype, and immunomodulators, as well as TIME-related prognosis, in LIHC. Furthermore, this study established six favorable prognostic models to predict LIHC prognosis based on the SOX4-associated genes, including DNA damage repair-related genes, EMT-related genes, M6A methylation-related genes, hypoxia-related genes, energy-metabolism-related genes, and ferroptosis-related genes. Finally, we found that SOX4 played an important role in the drug resistance of lenvatinib in LICH in vitro. Altogether, this study emphasizes the critical roles of SOX4 in the diagnosis and prognosis of tumors, especially in LIHC, and as a promising therapeutic target for tumor treatment.

## Figures and Tables

**Table 1:** Risk factors of *SOX4*-associated genes.

Variable	Model		
	β-Coefficient	Odds Ratio (95% CI)	*p*-Value
Immune-related genes			
CD244	0.509	1.664 (1.145, 2.420)	0.008
CD274	−0.52	0.594 (0.357, 0.988)	0.045
TGFB1	0.118	1.211 (1.008, 1.454)	0.041
CD27	−0.438	0.645 (0.453, 0.919)	0.015
IL2RA	0.523	1.687 (1.108, 2.569)	0.015
TMEM173	−0.606	0.545 (0.389, 0.765)	<0.001
TNFRSF4	0.331	1.392 (1.087, 1.784)	0.009
TNFSF4	0.212	1.236 (1.007, 1.518)	0.043
CD40	0.222	1.249 (1.046, 1.492)	0.014
TAP2	0.412	1.510 (1.025, 2.223)	0.037
DNA damage-related genes			
RFC3	−0.574	0.563 (0.415, 0.763)	<0.001
RAD54B	0.876	2.401 (1.006, 5.728)	0.048
MUTYH	0.407	1.503 (1.010, 2.236)	0.044
MGMT	−0.418	0.658 (0.509, 0.851)	0.001
HAP1	0.582	1.789 (1.232, 2.598)	0.002
UVSSA	−0.456	0.634 (0.411, 0.978)	0.039
EMT-related genes			
ACTA2	−0.235	0.791 (0.671, 0.933)	0.005
PHLDA2	0.186	1.204 (1.070, 1.355)	0.002
SGCB	0.262	1.299 (1.118, 1.509)	<0.001
NKX3-2	0.403	1.497 (1.059, 2.116)	0.022
M6A methylation-related genes			
ZC3H13	−0.456	0.634 (0.476, 0.843)	0.002
YTHDF2	0.669	1.951 (1.207, 3.154)	0.006
Hypoxia-related genes			
CUL2	0.405	1.500 (1.019, 2.208)	0.04
EPO	0.148	1.160 (1.059, 1.270)	0.001
UBB	−0.443	0.642 (0.450, 0.916)	0.014
Energy-metabolism-related genes			
GGT3P	2.596	13.410 (2.186, 82.250)	0.005
LDHA	0.305	1.356 (1.015, 1.813)	0.04
NQO2	0.236	1.266 (1.031, 1.556)	0.025
Ferroptosis-related genes			
SAT1	−0.283	1.115 (0.576, 0.986)	0.039
SLC7A11	0.192	1.212 (1.029, 1.426)	0.021
CISD1	0.354	1.425 (1.020, 1.992)	0.078

## Data Availability

All data for this study are available from TCGA and GEO databases. This study benefited from TCGA and GEO databases. We appreciate the data platform and the authors uploaded their data.

## Supplementary Materials

The following are available online at https://www.mdpi.com/article/10.3390/cancers15215235/s1, Figure S1: Representative immunohistochemical staining results of SOX4 in various types of tumors, Figure S2: Prognostic values of SOX4 determined by Kaplan-Meier Plotter, Figure S3: The correlation of SOX4 mutation count with the fraction of copy number altered genome, Figure S4: The correlation of SOX4 expression with TMB in different tumors, Figure S5: The correlation of SOX4 expression with MSI in different tumors, Figure S6: The correlation between SOX4 expression and the infiltration level of immune cells visualized by the cluster heatmaps, Figure S7: The correlation between SOX4 expression and the markers of immune and immune-related cells visualized by the cluster heatmap, Figure S8: The correlation between SOX4 expression and immune subtypes, molecular subtypes in different tumors, Figure S9: The correlation between SOX4 expression and three kinds of immunomodulators in different tumors visualized by the cluster heatmap, Figure S10: The correlation of SOX4 expression with immunoinhibitors visualized by the box plots, Figure S11: The correlation of SOX4 expression with immunostimulators visualized by the box plots, Figure S12: The correlation of SOX4 expression with MHC molecules visualized by the box plots, Figure S13: The multigene prognostic model based on SOX4 associated Immune-related genes to predict LIHC prognosis, Figure S14: The multigene prognostic model based on SOX4 associated DNA damage-related genes to predict LIHC prognosis, Figure S15: The multigene prognostic model based on SOX4 associated EMT-related genes to predict LIHC prognosis, Figure S16: The multigene prognostic model based on SOX4 associated hypoxia-related genes to predict LIHC prognosis, Figure S17: The multigene prognostic model based on SOX4 associated energy metabolism-related genes to predict LIHC prognosis, Figure S18: The multigene prognostic model based on SOX4 associated ferroptosis-related genes to predict LIHC prognosis.

## References

[1] Sung H., Ferlay J., Siegel R.L., Laversanne M., Soerjomataram I., Jemal A., Bray F. (2021). Global Cancer Statistics 2020: GLOBOCAN Estimates of Incidence and Mortality Worldwide for 36 Cancers in 185 Countries. CA Cancer J. Clin..

[2] Islami F., Ward E.M., Sung H., Cronin K.A., Tangka F.K.L., Sherman R.L., Zhao J., Anderson R.N., Henley S.J., Yabroff K.R. (2021). Annual Report to the Nation on the Status of Cancer, Part 1: National Cancer Statistics. J. Natl. Cancer Inst..

[3] Cao W., Chen H.D., Yu Y.W., Li N., Chen W.Q. (2021). Changing profiles of cancer burden worldwide and in China: A secondary analysis of the global cancer statistics 2020. Chin. Med. J..

[4] Hanieh H., Ahmed E.A., Vishnubalaji R., Alajez N.M. (2020). SOX4: Epigenetic regulation and role in tumorigenesis. Semin. Cancer Biol..

[5] Chen J., Ju H.L., Yuan X.Y., Wang T.J., Lai B.Q. (2016). SOX4 is a potential prognostic factor in human cancers: A systematic review and meta-analysis. Clin. Transl. Oncol..

[6] Jiang Y., Ding Q., Xie X., Libby R.T., Lefebvre V., Gan L. (2013). Transcription factors SOX4 and SOX11 function redundantly to regulate the development of mouse retinal ganglion cells. J. Biol. Chem..

[7] Huang J.L., Wang X.K., Liao X.W., Han C.Y., Yu T.D., Huang K.T., Yang C.K., Liu X.G., Yu L., Zhu G.Z. (2021). SOX4 as biomarker in hepatitis B virus-associated hepatocellular carcinoma. J. Cancer.

[8] Hasegawa S., Nagano H., Konno M., Eguchi H., Tomokuni A., Tomimaru Y., Asaoka T., Wada H., Hama N., Kawamoto K. (2016). A crucial epithelial to mesenchymal transition regulator, Sox4/Ezh2 axis is closely related to the clinical outcome in pancreatic cancer patients. Int. J. Oncol..

[9] Pan S., Bao D., Li Y., Liu D., Quan S., Wang R. (2022). SOX4 induces drug resistance of colorectal cancer cells by downregulating CYLD through transcriptional activation of microRNA-17. J. Biochem. Mol. Toxicol..

[10] Sogawa C., Eguchi T., Namba Y., Okusha Y., Aoyama E., Ohyama K., Okamoto K. (2021). Gel-Free 3D Tumoroids with Stem Cell Properties Modeling Drug Resistance to Cisplatin and Imatinib in Metastatic Colorectal Cancer. Cells.

[11] Song H.M., Meng D., Wang J.P., Zhang X.Y. (2021). circRNA hsa_circ_0005909 Predicts Poor Prognosis and Promotes the Growth, Metastasis, and Drug Resistance of Non-Small-Cell Lung Cancer via the miRNA-338-3p/SOX4 Pathway. Dis. Markers.

[12] Huang X., Qi L., Lu W., Li Z., Li W., Li F. (2021). MYCN contributes to the sensitization of acute myelogenous leukemia cells to cisplatin by targeting SRY-box transcription factor 4. Bioengineered.

[13] Cheng Y., Zhan P., Lu J., Lu Y., Luo C., Cen X., Wang F., Xie C., Yin Z. (2023). Metformin synergistically enhances the antitumour activity of Lenvatinib in hepatocellular carcinoma by altering AKT-FOXO3 signalling pathway. Liver Int..

[14] Tarabichi M., Demeulemeester J., Verfaillie A., Flanagan A.M., Van Loo P., Konopka T. (2021). A pan-cancer landscape of somatic mutations in non-unique regions of the human genome. Nat. Biotechnol..

[15] Zhang J., Jiang H., Du K., Xie T., Wang B., Chen C., Reiter R.J., Cen B., Yuan Y. (2021). Pan-cancer analyses reveal genomics and clinical characteristics of the melatonergic regulators in cancer. J. Pineal Res..

[16] Kulkarni A., Chang M.T., Vissers J.H.A., Dey A., Harvey K.F. (2020). The Hippo Pathway as a Driver of Select Human Cancers. Trends Cancer.

[17] Ganini C., Amelio I., Bertolo R., Bove P., Buonomo O.C., Candi E., Cipriani C., Di Daniele N., Juhl H., Mauriello A. (2021). Global mapping of cancers: The Cancer Genome Atlas and beyond. Mol. Oncol..

[18] Moreno C.S. (2020). SOX4: The unappreciated oncogene. Semin. Cancer Biol..

[19] Zheng Y., Zhang J., Ye B. (2020). miR-138 mediates sorafenib-induced cell survival and is associated with poor prognosis in cholangiocarcinoma cells. Clin. Exp. Pharmacol. Physiol..

[20] Lu J.W., Hsieh M.S., Hou H.A., Chen C.Y., Tien H.F., Lin L.I. (2017). Overexpression of SOX4 correlates with poor prognosis of acute myeloid leukemia and is leukemogenic in zebrafish. Blood Cancer J..

[21] Teimouri H., Kolomeisky A.B. (2021). Temporal order of mutations influences cancer initiation dynamics. Phys. Biol..

[22] Giorgi G., Del Re B. (2021). Epigenetic dysregulation in various types of cells exposed to extremely low-frequency magnetic fields. Cell Tissue Res..

[23] Su K., Wang N., Shao Q., Liu H., Zhao B., Ma S. (2021). The role of a ceRNA regulatory network based on lncRNA MALAT1 site in cancer progression. Biomed. Pharmacother..

[24] Wang J., Tang Q., Lu L., Luo Z., Li W., Lu Y., Pu J. (2019). LncRNA OIP5-AS1 interacts with miR-363-3p to contribute to hepatocellular carcinoma progression through up-regulation of SOX4. Gene Ther..

[25] Chen X., Zhang L., Zhang T., Hao M., Zhang X., Zhang J., Xie Q., Wang Y., Guo M., Zhuang H. (2013). Methylation-mediated repression of microRNA 129-2 enhances oncogenic SOX4 ex-pression in HCC. Liver Int..

[26] Zhang J., Ke S., Zheng W., Zhu Z., Wu Y. (2020). Hsa_circ_0003645 Promotes Breast Cancer Progression by Regulating miR-139-3p/HMGB1 Axis. Oncol. Targets Ther..

[27] Xu J., Guo J., Jiang Y., Liu Y., Liao K., Fu Z., Xiong Z. (2019). Improved characterization of the relationship between long intergenic non-coding RNA Linc00152 and the occurrence and development of malignancies. Cancer Med..

[28] Yang Y., Wang Y. (2021). Role of Epigenetic Regulation in Plasticity of Tumor Immune Microenvironment. Front. Immunol..

[29] Huang R., Zhou P.K. (2021). DNA damage repair: Historical perspectives, mechanistic pathways and clinical translation for tar-geted cancer therapy. Signal Transduct. Target. Ther..

[30] Lachat C., Peixoto P., Hervouet E. (2021). Epithelial to Mesenchymal Transition History: From Embryonic Development to Cancers. Biomolecules.

[31] Sun T., Wu R., Ming L. (2019). The role of m6A RNA methylation in cancer. Biomed. Pharmacother..

[32] Jing X., Yang F., Shao C., Wei K., Xie M., Shen H., Shu Y. (2019). Role of hypoxia in cancer therapy by regulating the tumor micro-environment. Mol. Cancer.

[33] Schömel N., Geisslinger G., Wegner M.S. (2020). Influence of glycosphingolipids on cancer cell energy metabolism. Prog. Lipid Res..

[34] Wang H., Cheng Y., Mao C., Liu S., Xiao D., Huang J., Tao Y. (2021). Emerging mechanisms and targeted therapy of ferroptosis in cancer. Mol. Ther..

[35] Guo J., Zhao J., Xu Q., Huang D. (2022). Resistance of Lenvatinib in Hepatocellular Carcinoma. Curr. Cancer Drug Targets.

