# Supplementary material for: Pan-Cancer Analysis and Experimental Validation of SOX4 as a Potential Diagnosis, Prognosis, and Immunotherapy Biomarker
Source: Cancers (Basel). 2023 Oct 31;15(21):5235. doi: 10.3390/cancers15215235 (PMC10649301; doi:10.3390/cancers15215235)

Supplementary Material

1 Supplementary Figures and Tables

1.1 Supplementary Figures

Supplementary Figure 1. Representative immunohistochemical staining results of *SOX4* in various types of tumors. (a) Glioma (b) Thyroid cancer (c) Lung cancer (d) Colorectal cancer (e) Head and neck cancer (f) Stomach cancer (g) Liver cancer (h) Carcinoid (i) Pancreatic cancer (j) Renal cancer (k) Urothelial cancer (l) Prostate cancer (m) Testis cancer (n) Breast cancer (o) Cervical cancer (p) Endometrial cancer (q) Ovarian cancer (r) Melanoma (s) Skin cancer (t) Lymphoma

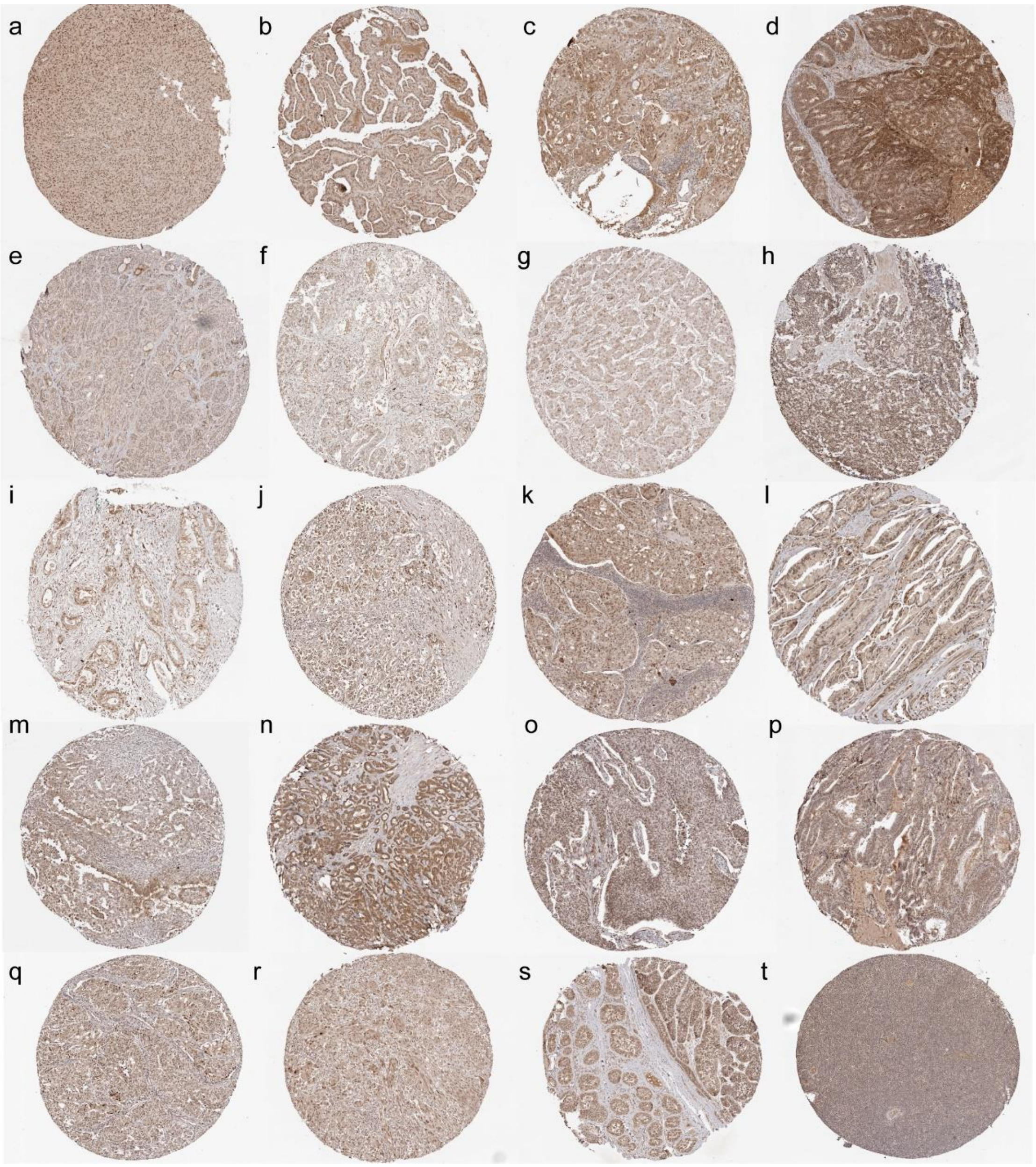

Supplementary Figure 2. Prognostic values of *SOX4* determined by Kaplan-Meier Plotter. (a) The correlation of *SOX4* expression with OS. (b) The correlation of *SOX4* expression with DFS.

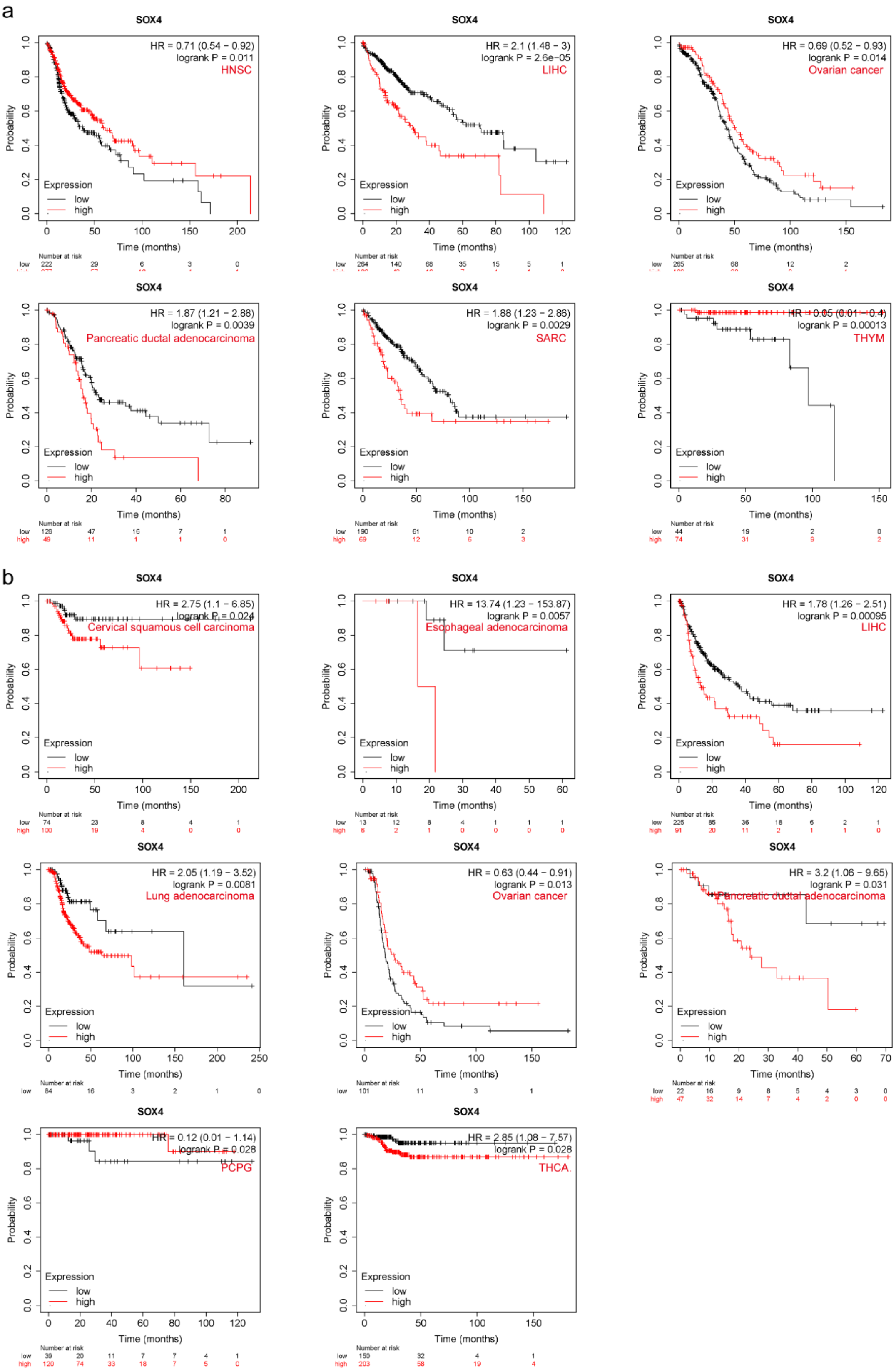

Supplementary Figure 3. The correlation of *SOX4* mutation count with the fraction of copy number altered genome.

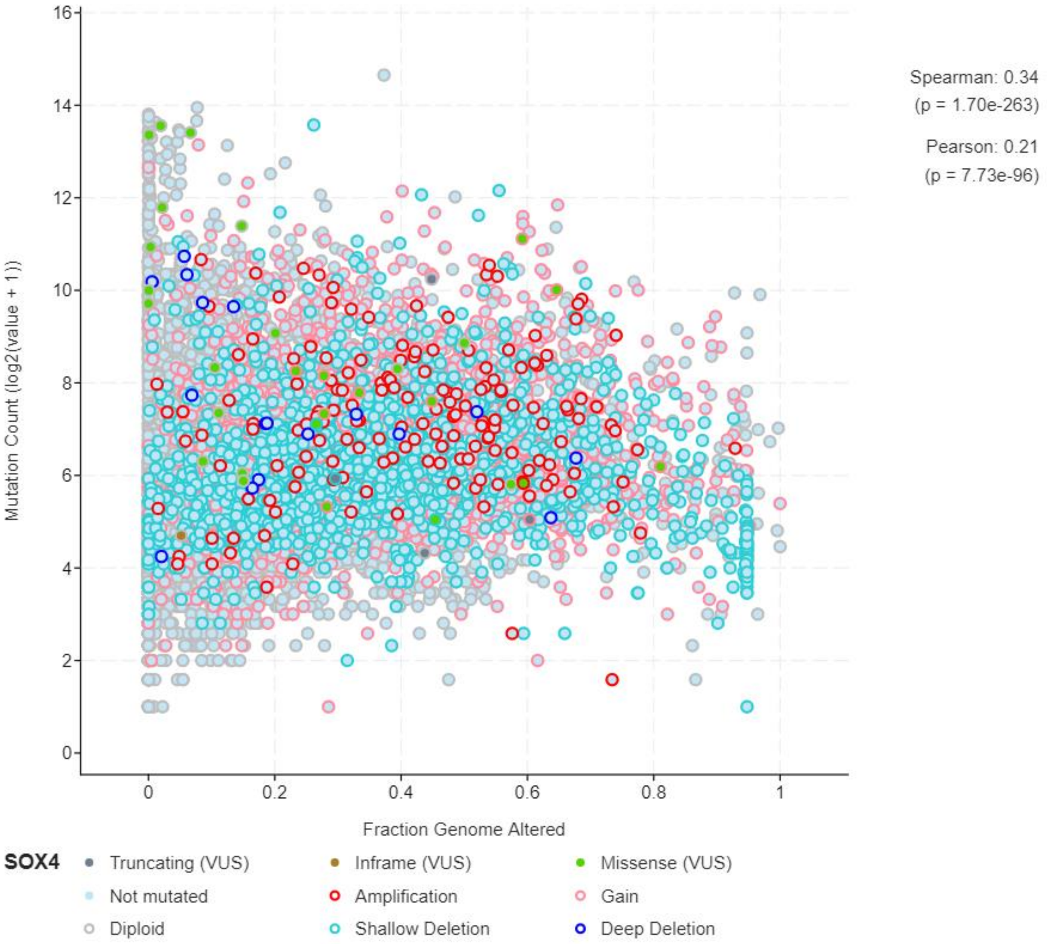

Supplementary Figure 4. The correlation of *SOX4* expression with TMB in different tumors.

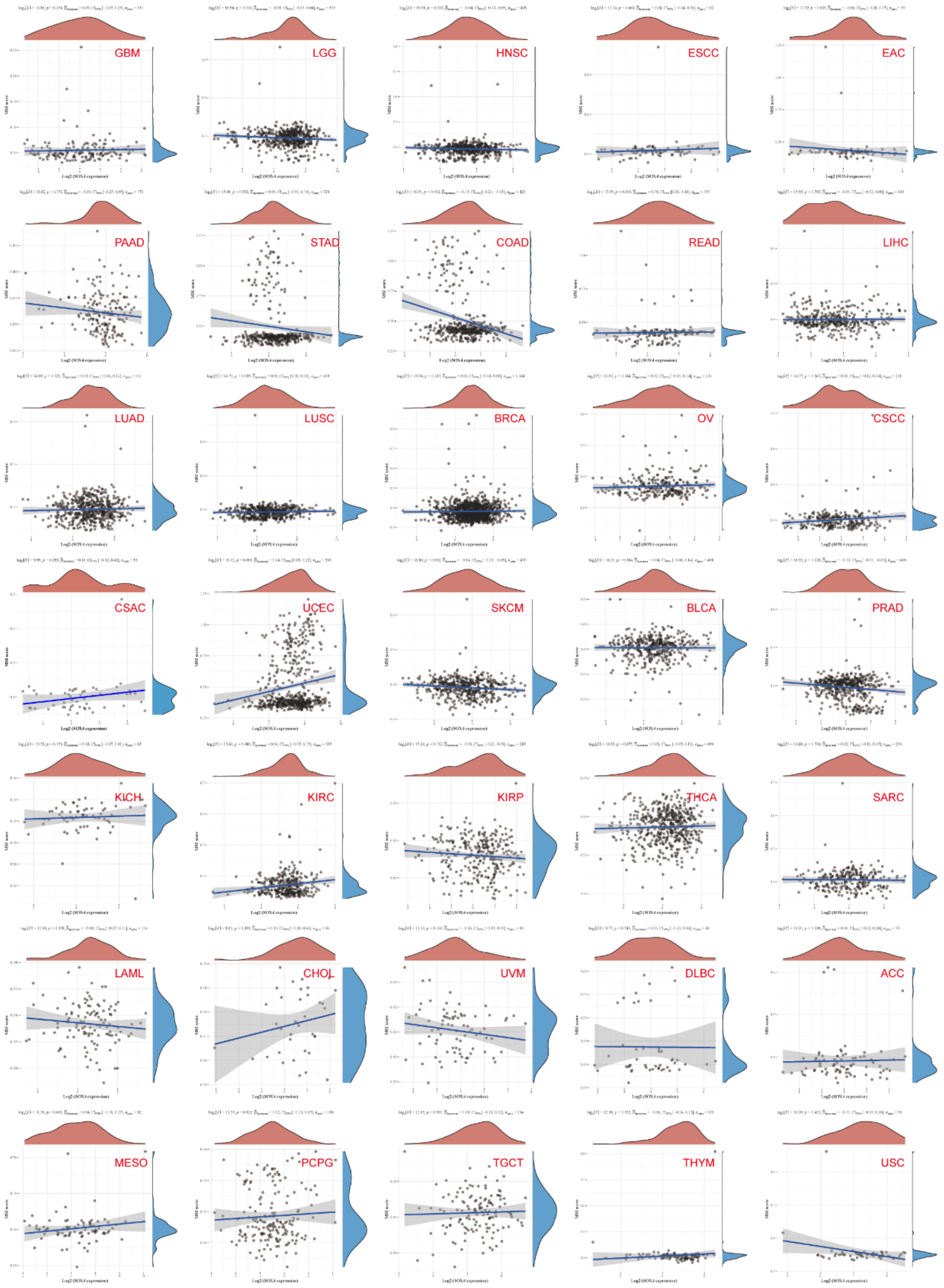

Supplementary Figure 5. The correlation of *SOX4* expression with MSI in different tumors.

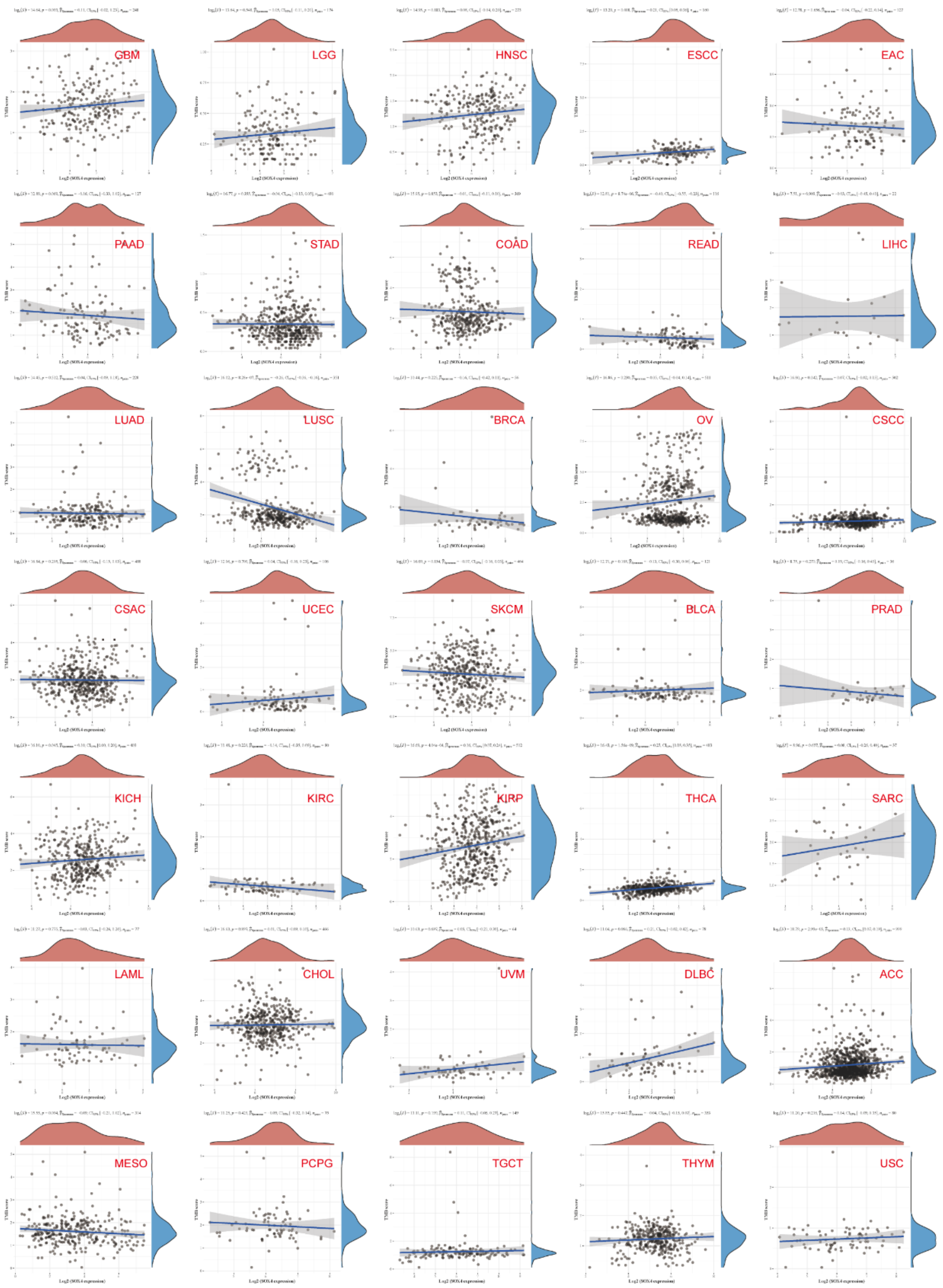

Supplementary Figure 6. The correlation between *SOX4* expression and the infiltration level of immune cells visualized by the cluster heatmaps.

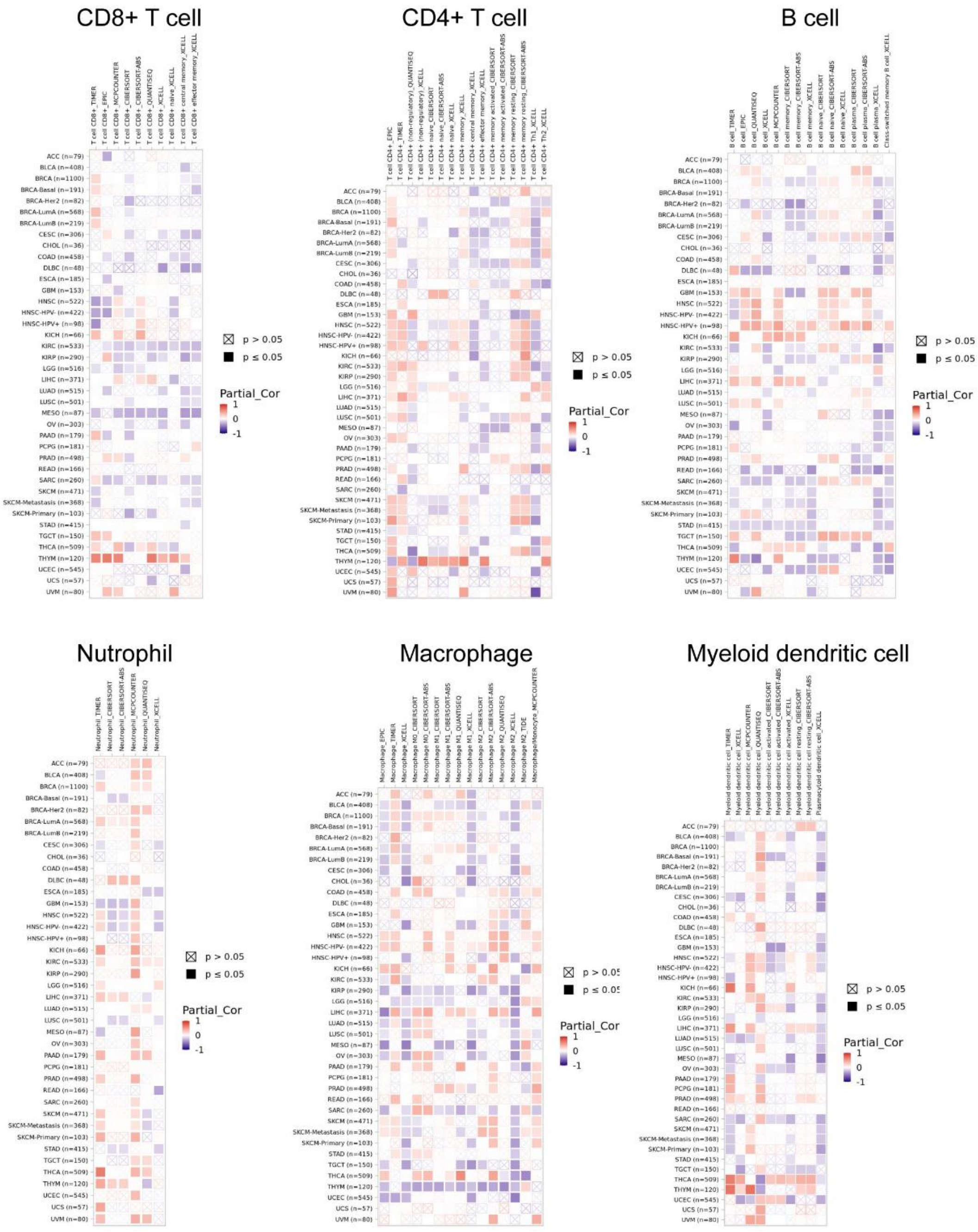

Supplementary Figure 7. The correlation between *SOX4* expression and the markers of immune and immune-related cells visualized by the cluster heatmap.

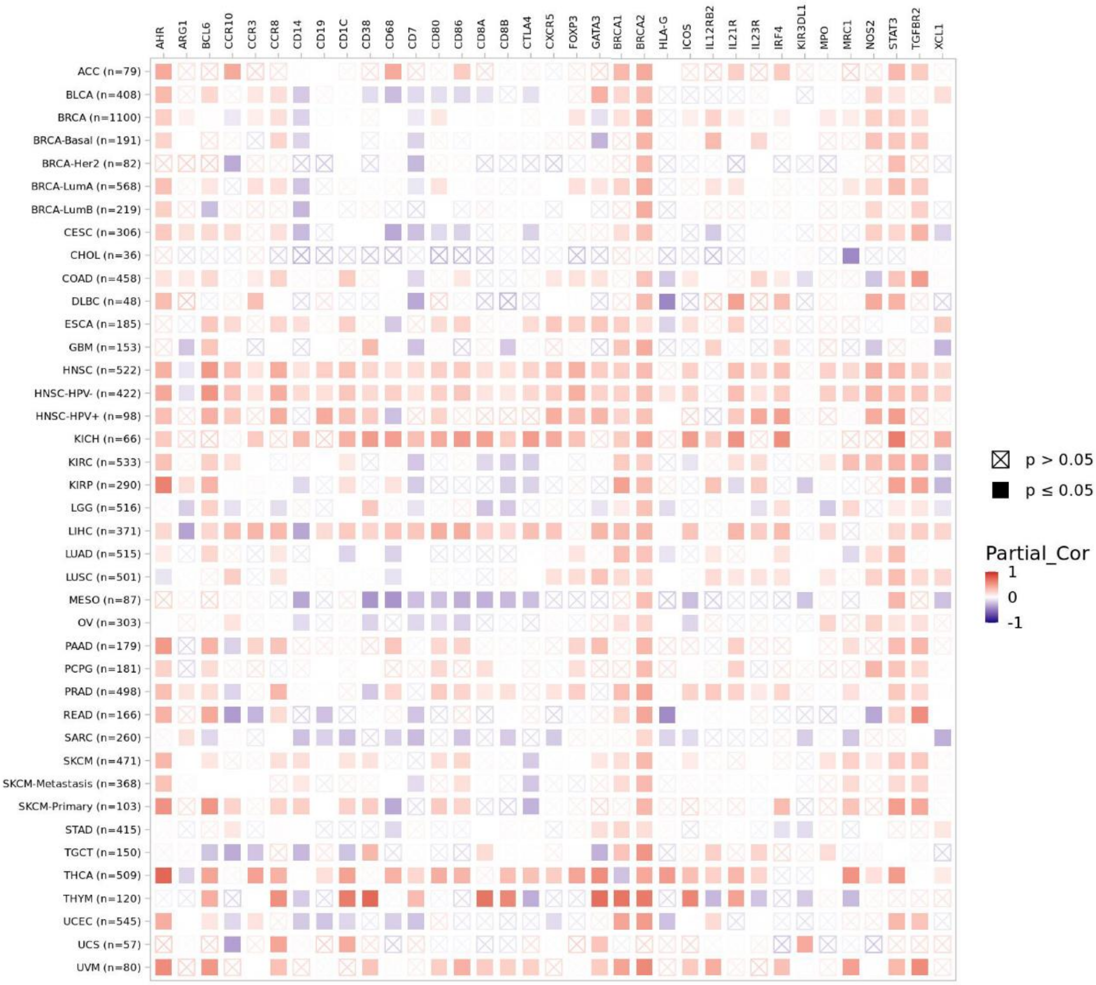

Supplementary Figure 8. The correlation between *SOX4* expression and immune subtypes, molecular subtypes in different tumors.

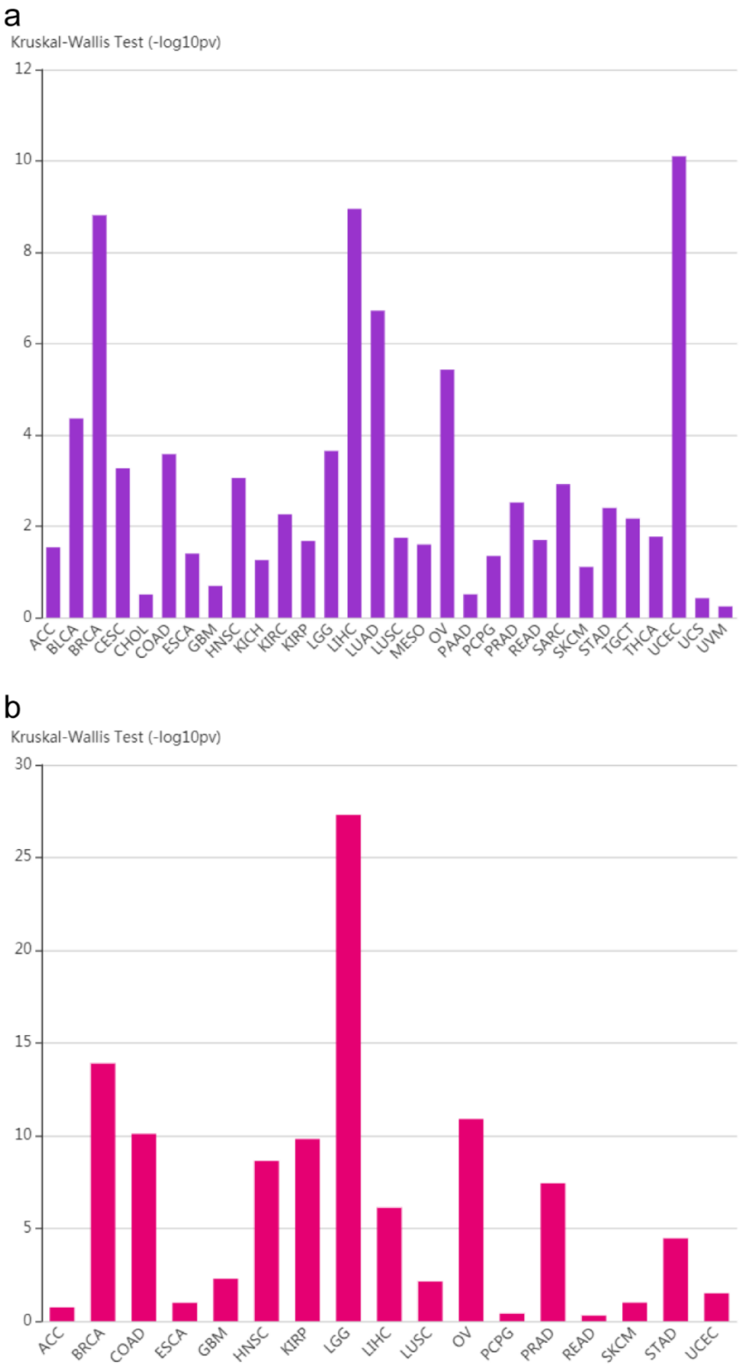

Supplementary Figure 9. The correlation between *SOX4* expression and three kinds of immunomodulators in different tumors visualized by the cluster heatmap. (a) The correlation of *SOX4* expression with immunoinhibitors. (b) The correlation of *SOX4* expression with immunostimulators. (c) The correlation of *SOX4* expression with MHC molecules.

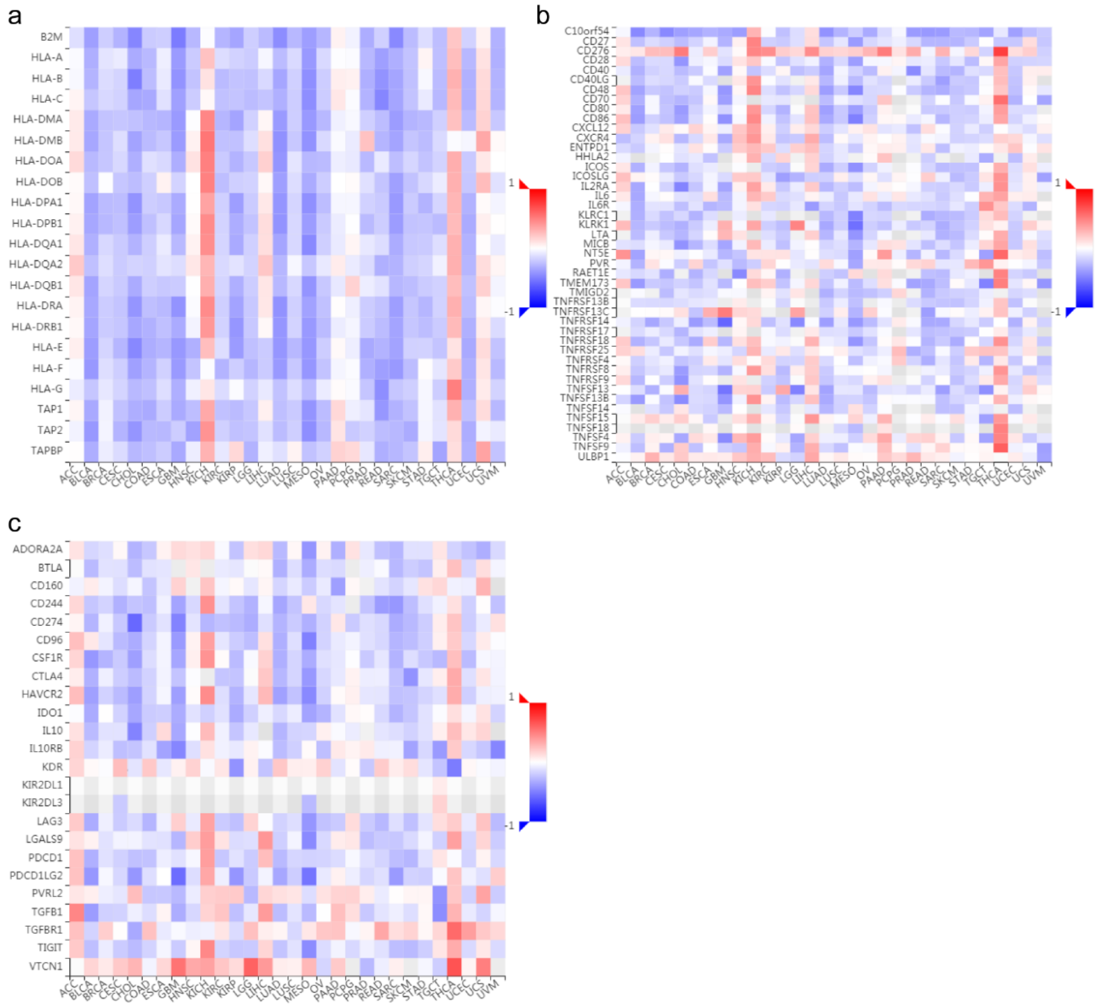

Supplementary Figure 10. The correlation of *SOX4* expression with immunoinhibitors visualized by the box plots.

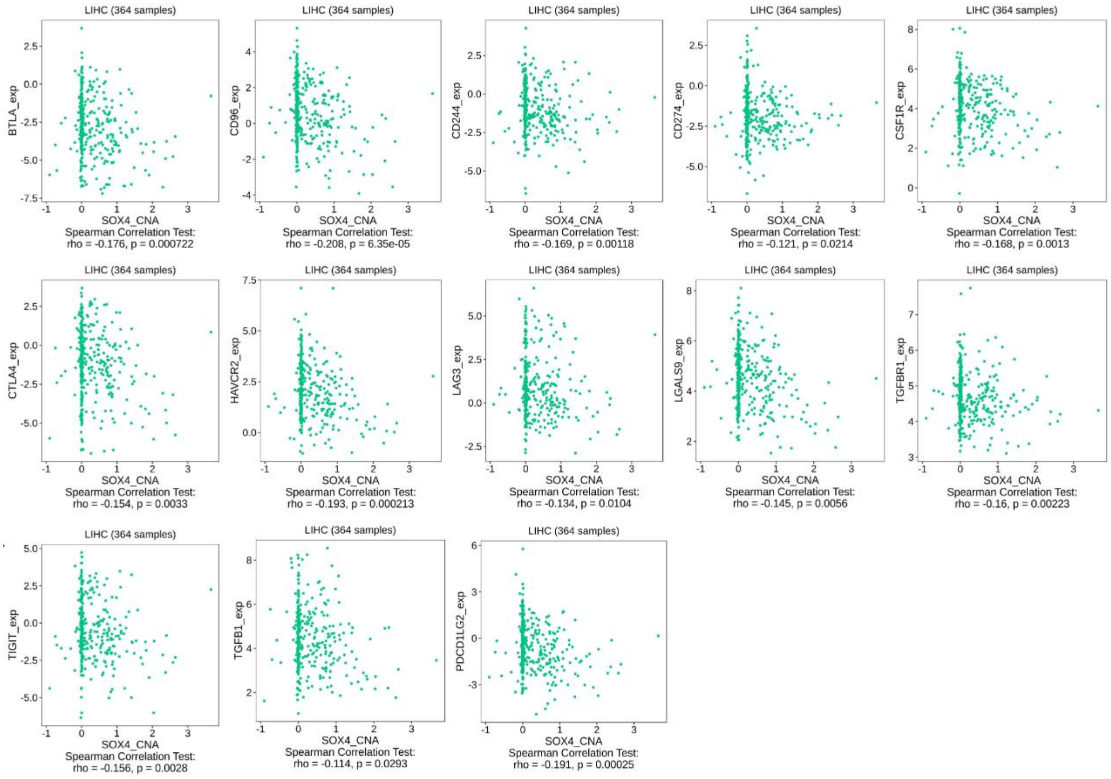

Supplementary Figure 11. The correlation of *SOX4* expression with immunostimulators visualized by the box plots.

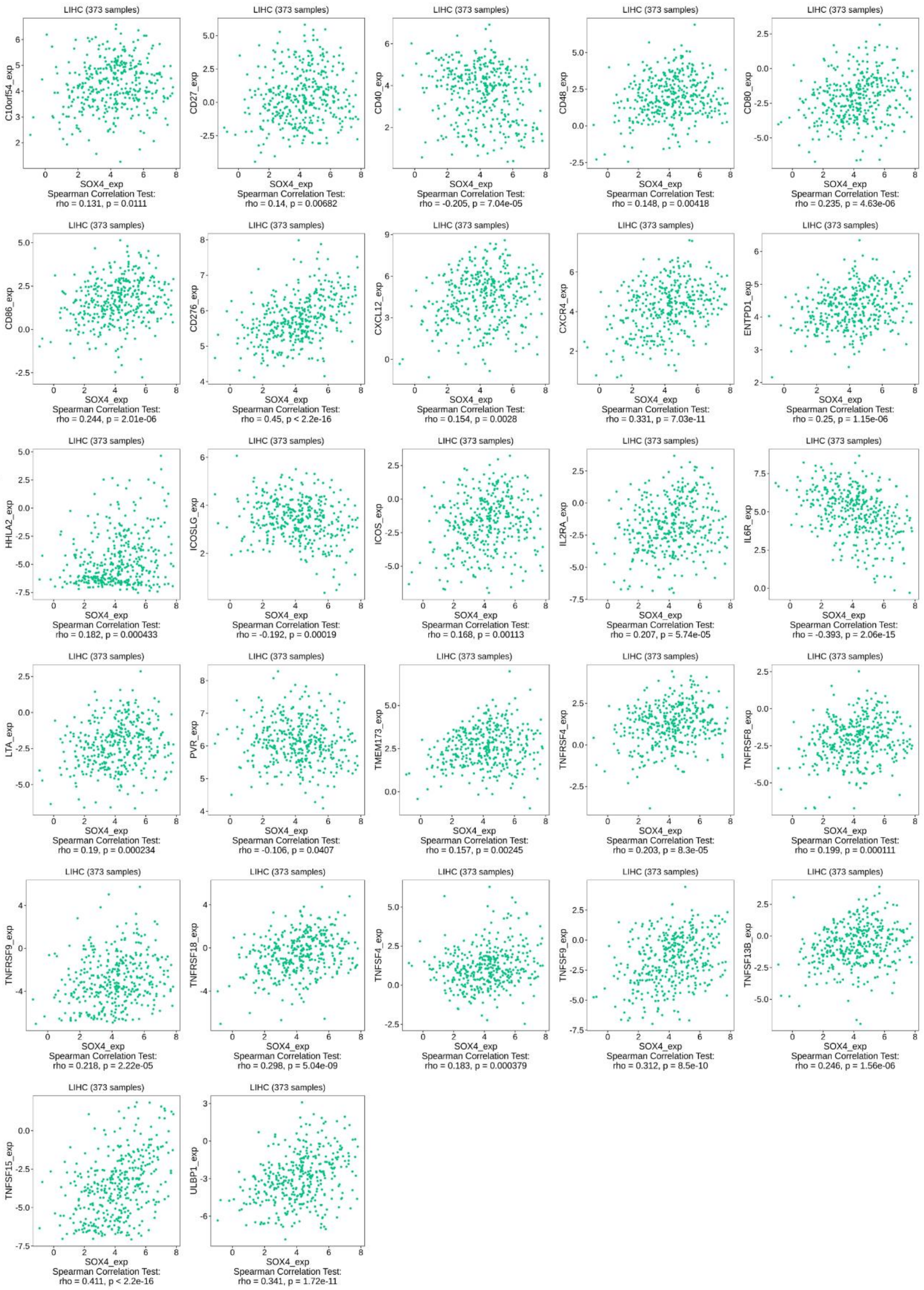

Supplementary Figure 12. The correlation of *SOX4* expression with MHC molecules visualized by the box plots.

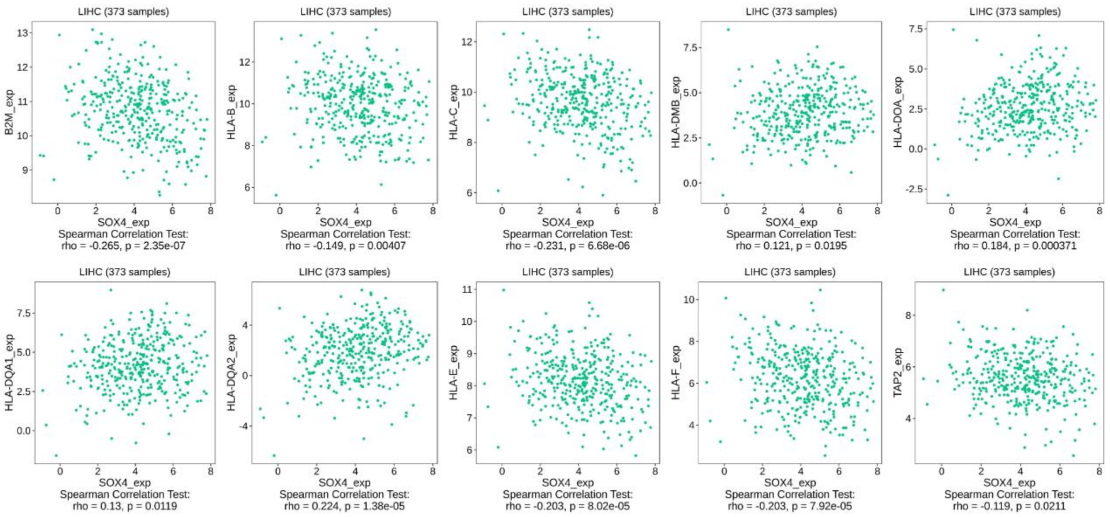

Supplementary Figure 13. The multigene prognostic model based on *SOX4* associated immune-related genes to predict LIHC prognosis. (a) LASSO coefficient profiles of ten features. (b) Identification of the optimal penalization coefficient lambda (λ) in the LASSO model with 10-fold cross-validation. (c)The curve of risk score. (d)The survival time and survival status of patients. (e) The expression profiles of ten prognostic genes visualized by the cluster heatmap. (f) Kaplan-Meier analysis for patient OS with high- and low-risk groups. (g)Time-dependent ROC analysis at 1-, 3-, and 5-year survival.

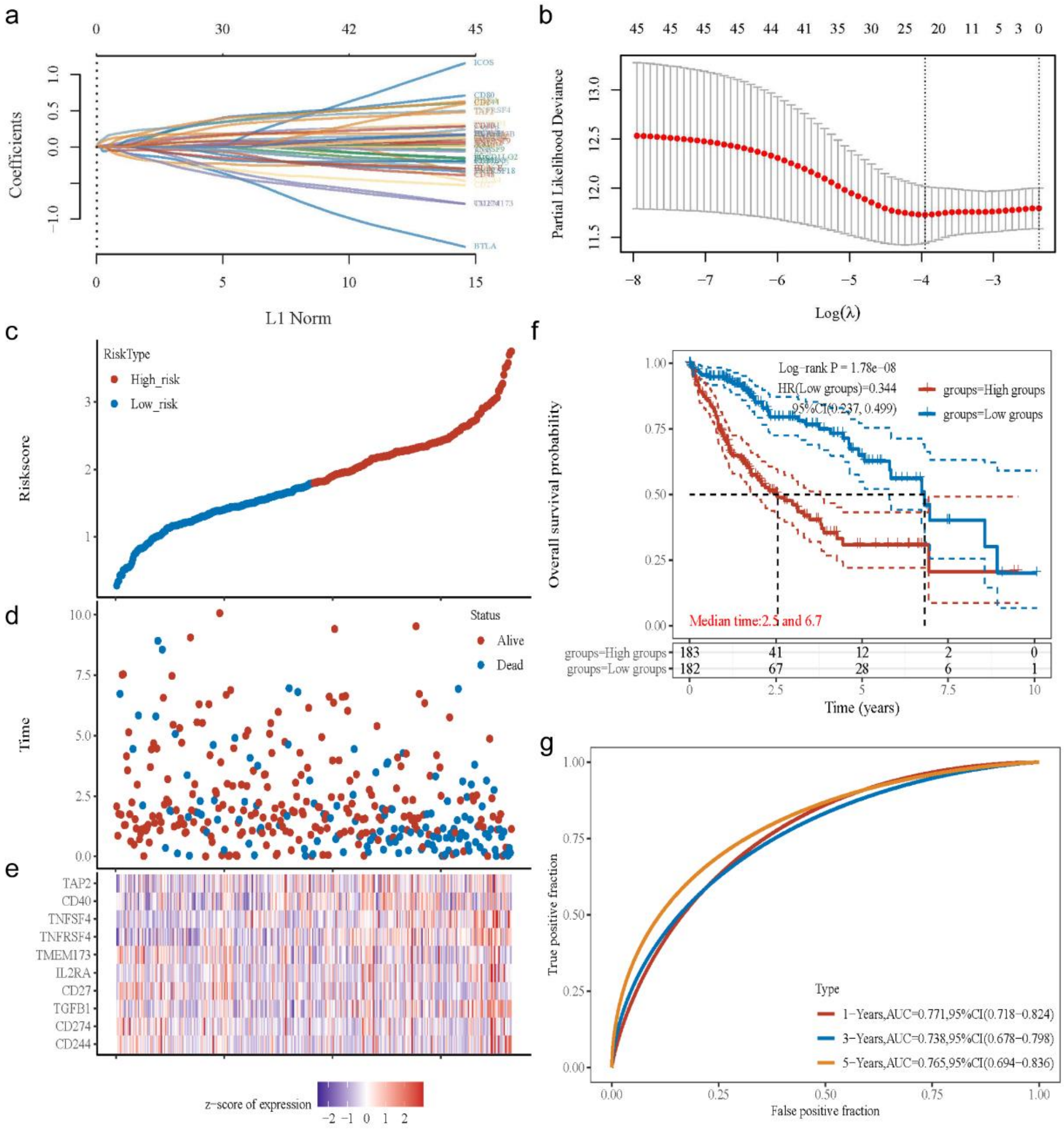

Supplementary Figure 14. The multigene prognostic model based on *SOX4* associated DNA damage-related genes to predict LIHC prognosis. (a) The cluster heatmaps. (b) LASSO coefficient profiles of six features. (c) Identification of the optimal penalization coefficient lambda (λ) in the LASSO model with 10-fold cross-validation. (d) The curve of risk score. (e) The survival time and survival status of patients. (f) The expression profiles of six prognostic genes visualized by the cluster heatmap. (g) Kaplan-Meier analysis for patient OS with high- and low-risk groups. (h) Time-dependent ROC analysis at 1-, 3-, and 5-year survival.

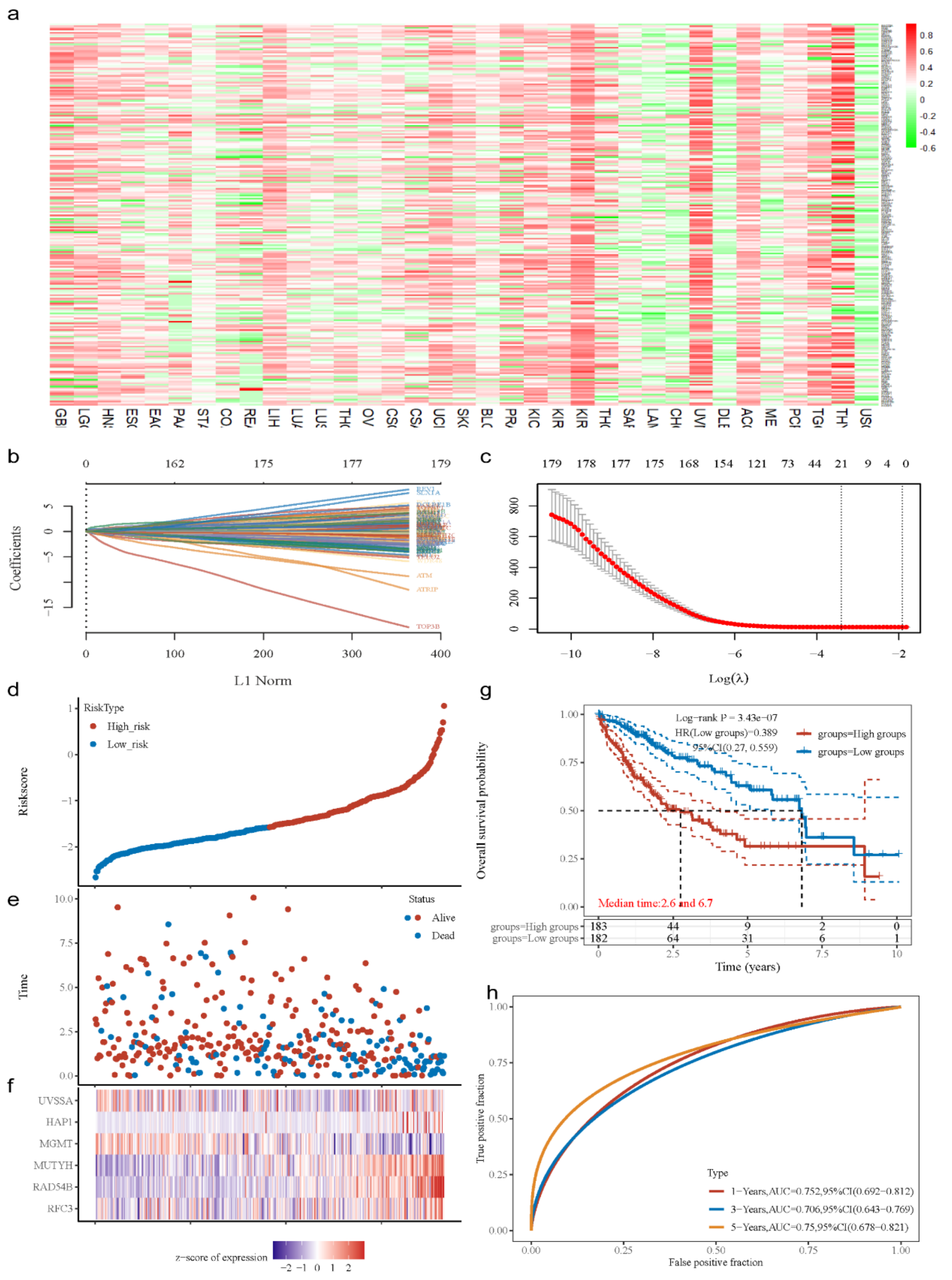

Supplementary Figure 15. The multigene prognostic model based on *SOX4* associated EMT-related genes to predict LIHC prognosis. (a)The cluster heatmaps. (b) LASSO coefficient profiles of two features. (c) Identification of the optimal penalization coefficient lambda (λ) in the LASSO model with 10-fold cross-validation. (d)The curve of risk score. (e)The survival time and survival status of patients. (f) The expression profiles of two prognostic genes visualized by the cluster heatmap. (g) Kaplan-Meier analysis for patient OS with high- and low-risk groups. (h)Time-dependent ROC analysis at 1-, 3-, and 5-year survival.

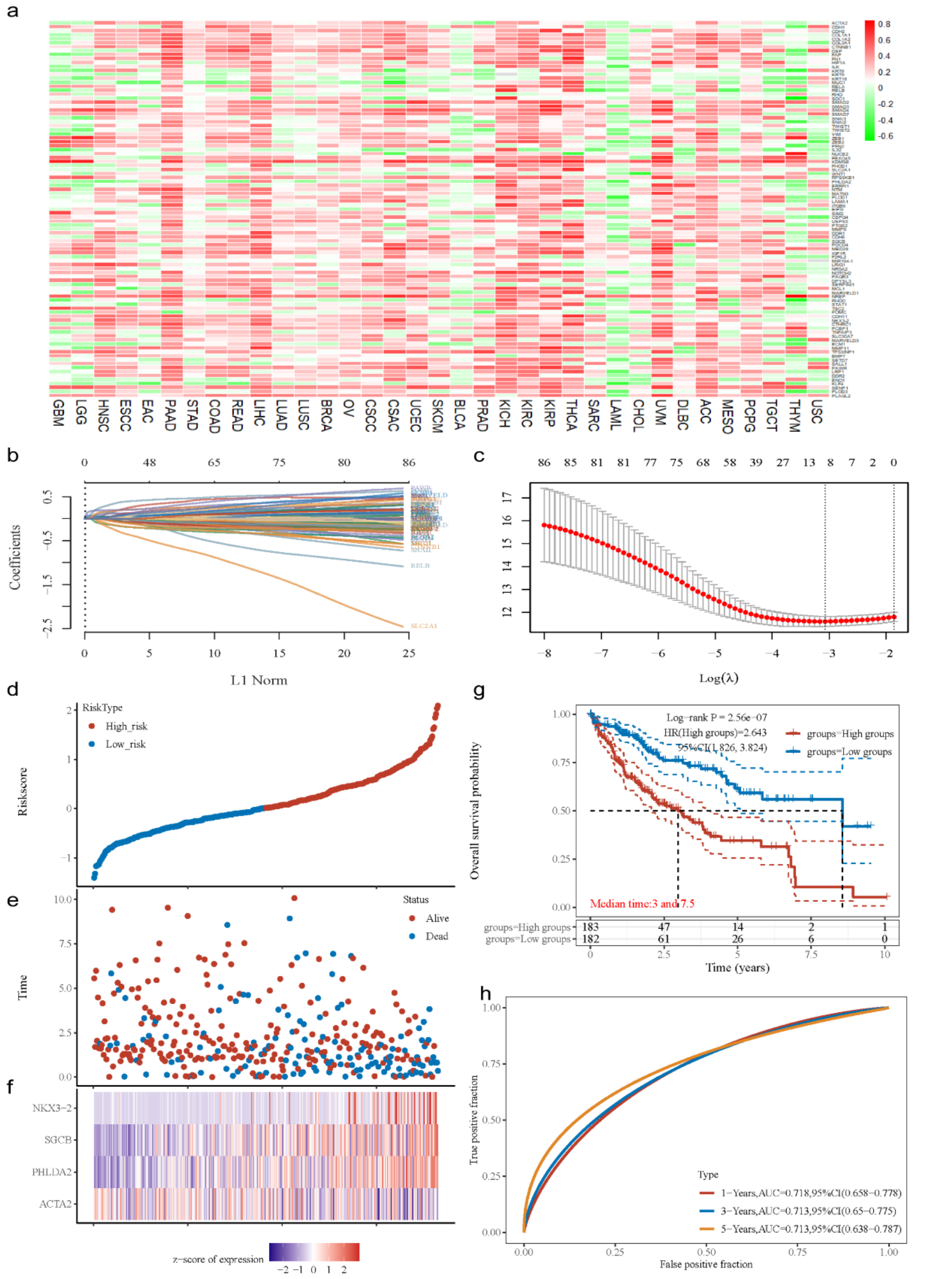

Supplementary Figure 16. The multigene prognostic model based on *SOX4* associated hypoxia-related genes to predict LIHC prognosis. (a)The cluster heatmaps. (b) LASSO coefficient profiles of three features. (c) Identification of the optimal penalization coefficient lambda (λ) in the LASSO model with 10-fold cross-validation. (d)The curve of risk score. (e)The survival time and survival status of patients. (f) The expression profiles of three prognostic genes visualized by the cluster heatmap. (g) Kaplan-Meier analysis for patient OS with high- and low-risk groups. (h)Time-dependent ROC analysis at 1-, 3-, and 5-year survival.

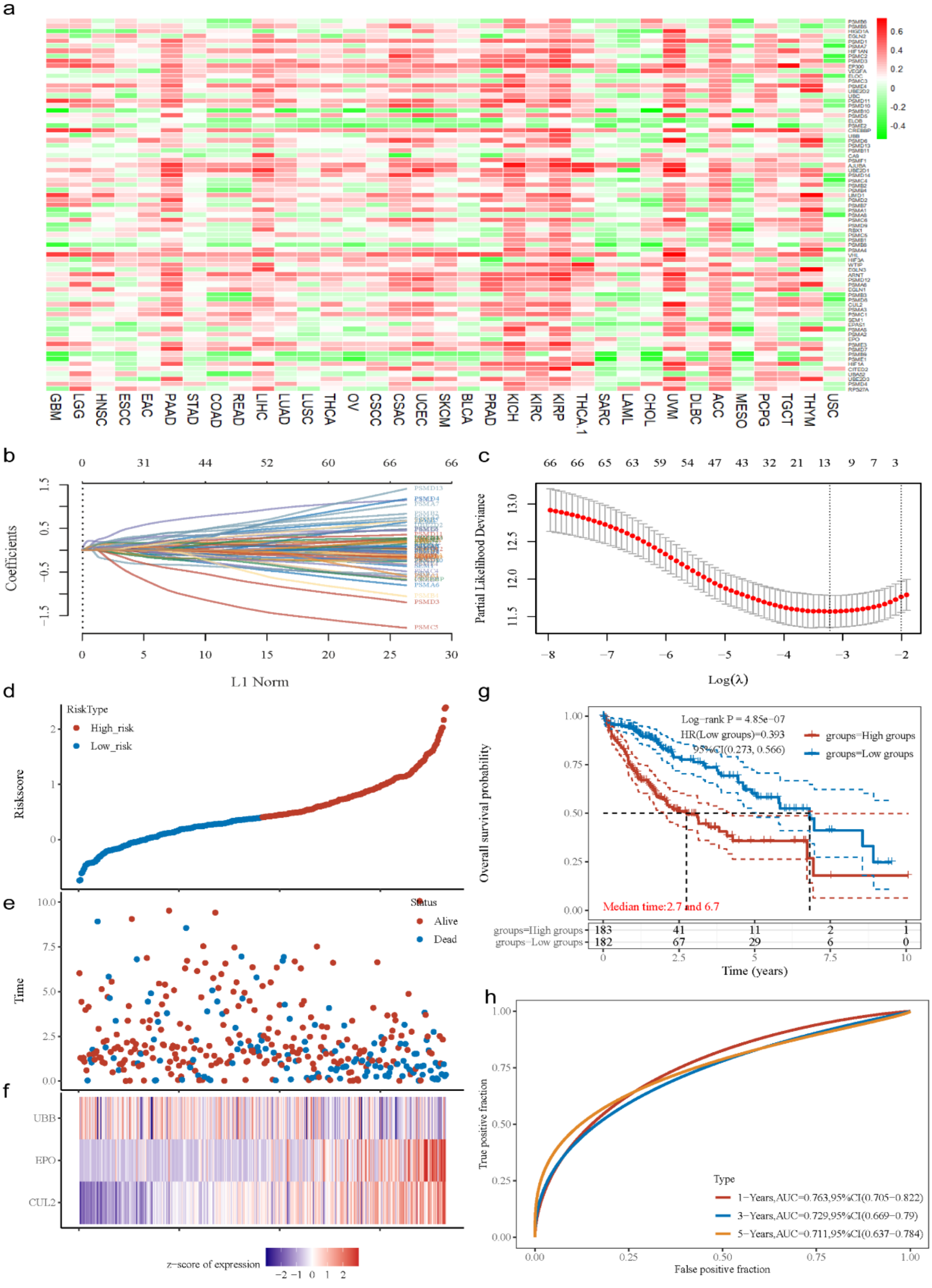

Supplementary Figure 17. The multigene prognostic model based on *SOX4* associated energy metabolism-related genes to predict LIHC prognosis. (a)The cluster heatmaps. (b) LASSO coefficient profiles of three features. (c) Identification of the optimal penalization coefficient lambda (λ) in the LASSO model with 10-fold cross-validation. (d)The curve of risk score. (e)The survival time and survival status of patients. (f) The expression profiles of three prognostic genes visualized by the cluster heatmap. (g) Kaplan-Meier analysis for patient OS with high- and low-risk groups. (h)Time-dependent ROC analysis at 1-, 3-, and 5-year survival.

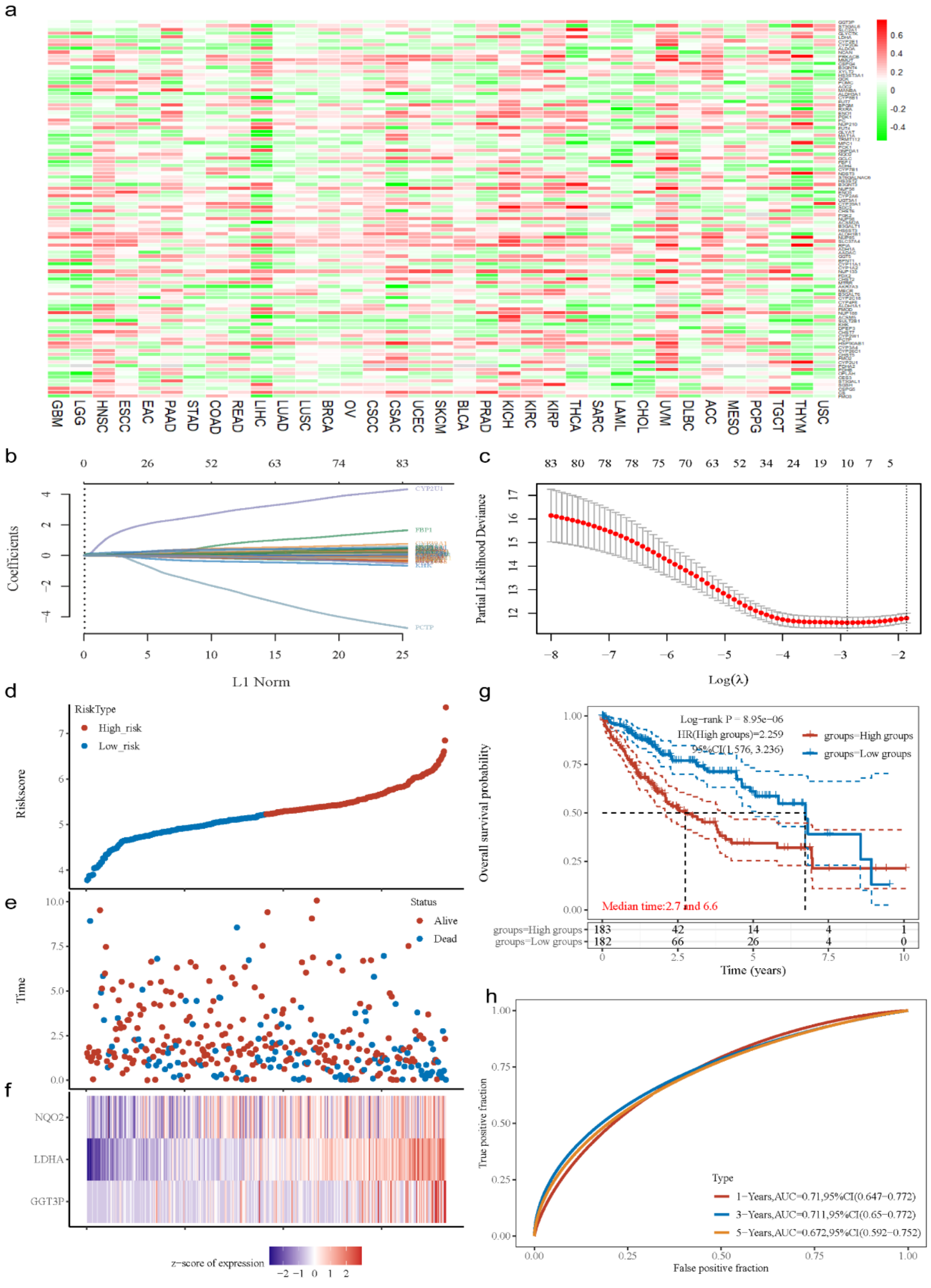

Supplementary Figure 18. The multigene prognostic model based on *SOX4* associated ferroptosis-related genes to predict LIHC prognosis. (a)The cluster heatmaps. (b) LASSO coefficient profiles of three features. (c) Identification of the optimal penalization coefficient lambda (λ) in the LASSO model with 10-fold cross-validation. (d)The curve of risk score. (e)The survival time and survival status of patients. (f) The expression profiles of three prognostic genes visualized by the cluster heatmap. (g) Kaplan-Meier analysis for patient OS with high- and low-risk groups. (h)Time-dependent ROC analysis at 1-, 3-, and 5-year survival.

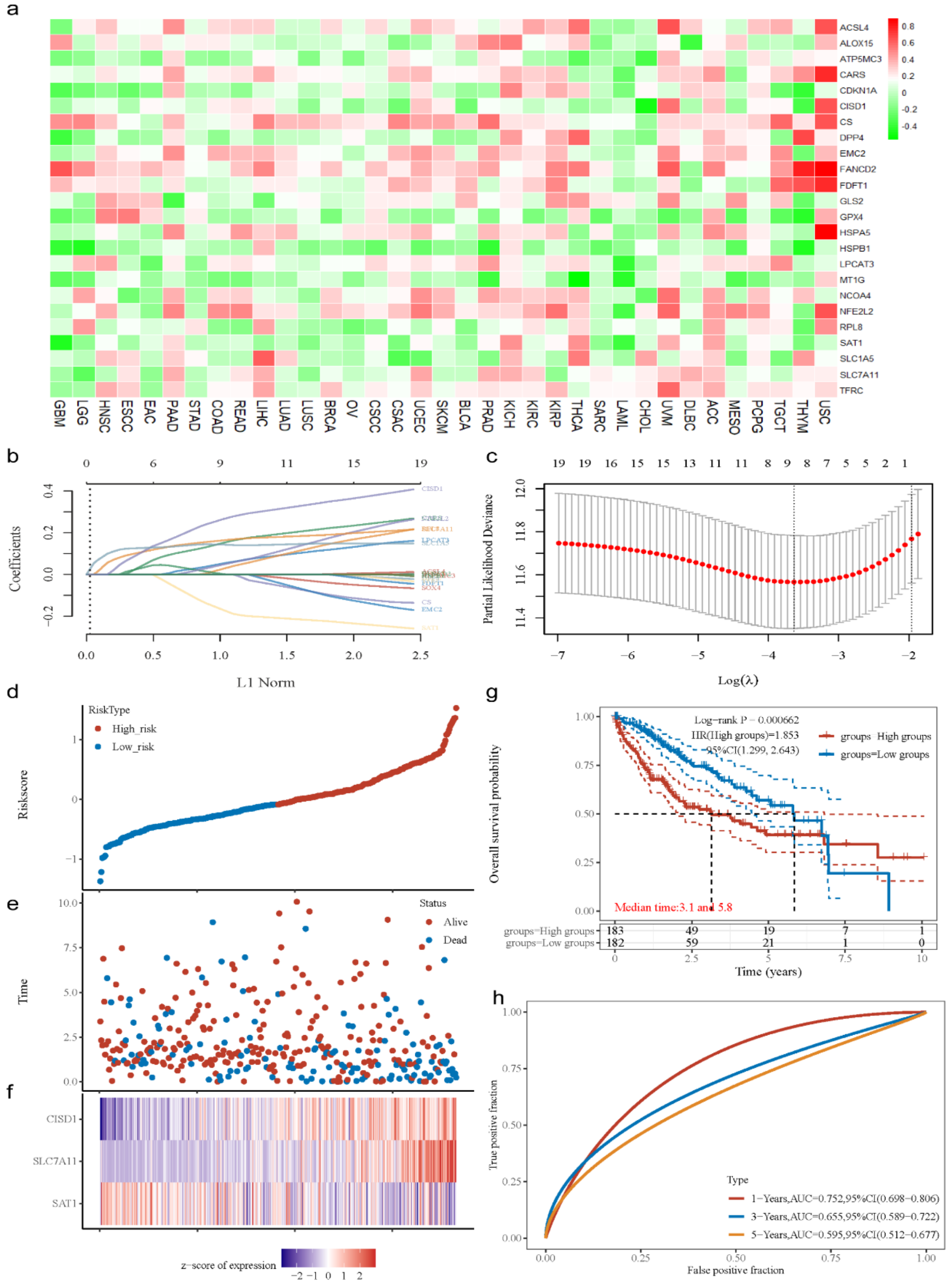

Supplement: Supplementary file 1 [file cancers-15-05235-s001.zip › cancers-2617833-supplementary.pdf]
